# The Pharyngeal Resistance Index—A Promising Diagnostic Tool in OSA

**DOI:** 10.1111/jsr.70341

**Published:** 2026-04-13

**Authors:** Nils Lucca Kern, Alexander Pugachev, Tobias Ebker, Tim Lukas Elter, Jan Oliver Voß, Axel Bumann, Max Heiland, Simon Bigus

**Affiliations:** ^1^ Department of Oral and Maxillofacial Surgery Charité – Universitätsmedizin Berlin, Corporate Member of Freie Universität Berlin and Humboldt‐Universität Zu Berlin Berlin Germany; ^2^ Simq GmbH Grafing bei München Germany; ^3^ MKG am Kaiserdamm Berlin Germany; ^4^ Mesantis 3D Dental‐Radiologicum GmbH Berlin Germany

**Keywords:** airway analysis, apnea‐hypopnea index, computational fluid dynamics, Maxillomandibular advancement, obstructive sleep apnea, pharyngeal resistance index

## Abstract

This study investigates the newly developed pharyngeal resistance index derived from computational fluid dynamics analysis of the upper airway in patients with obstructive sleep apnea. The aim was to evaluate this index as a diagnostic and severity assessment tool by comparing it with the apnea‐hypopnea index before and after maxillomandibular advancement surgery. A retrospective single‐center study was conducted at the Department of Oral and Maxillofacial Surgery, Charité—Universitätsmedizin Berlin, including 16 patients with obstructive sleep apnea who underwent maxillomandibular advancement between November 2019 and June 2023. Each patient received polysomnography and cone beam computed tomography preoperatively and at least three months postoperatively following a standardised protocol. The apnea‐hypopnea index and pharyngeal resistance index were statistically compared. The mean apnea‐hypopnea index decreased from 23.42 (SD = 22.53) preoperatively to 4.32 (SD = 5.0) postoperatively, a mean reduction of 81.55%. Mean pharyngeal resistance index decreased from 0.96 (SD = 0.81) preoperatively to 0.17 (SD = 0.22) postoperatively, representing an 82.29% reduction. Wilcoxon signed‐rank tests confirmed significant postoperative improvements for both indices (*p* < 0.01). Spearman analysis revealed no significant correlation between both indices, either preoperatively (ρ = 0.130, *p* = 0.633) or postoperatively (ρ = 0.159, *p* = 0.556). Class‐based comparison demonstrated improved agreement postoperatively, with most discrepancies limited to within ±1 class. Although no direct correlation was found, the pharyngeal resistance index demonstrated significant postoperative improvement and enhanced class‐level agreement with the apnea‐hypopnea index, supporting its future role as a supplementary diagnostic and predictive tool in obstructive sleep apnea management.

## 1 Introduction

Obstructive sleep apnea (OSA) is the most common sleep‐related breathing disorder, with a global prevalence of approximately 54%. Significant risk factors for OSA include a higher body mass index (BMI), older age, male gender, smoking, and alcohol consumption (de Araujo Dantas et al. 2023).

OSA is characterised by recurrent episodes of partial or complete upper airway (UA) obstruction during sleep leading to intermittent apnea, hypopnea, fragmented sleep and hypoxemia (Semelka et al. 2016). Typical symptoms include snoring or choking during sleep, excessive daytime sleepiness, fatigue, morning headaches, and cognitive impairments. OSA diagnosis and management are critical due to the associated health risks including increased risk of cardiovascular disease, metabolic disorders, depression, perioperative challenges and a possible link to a higher cancer risk (Semelka et al. 2016; Gawrys et al. 2024; Sun et al. 2022; Wang et al. 2025).

The diagnosis of OSA typically begins with a clinical evaluation, supplemented by validated screening tools like the STOP‐BANG or the Epworth Sleepiness Scale. Attended polysomnography (PSG) remains the gold standard approach, quantifying apnea and hypopnea events to calculate an apnea‐hypopnea index (AHI) (Malhotra et al. 2018; Kapur et al. 2017), a critical measure of disease severity. However, while the AHI is widely utilised, it has notable limitations in its accuracy in localising and characterising the anatomical and functional origins of airway obstruction. Drug‐Induced Sleep Endoscopy (DISE) as an additional diagnostic procedure tackles these limitations. While sedated, a flexible endoscope is inserted to visualise airway collapse in patients with OSA. It helps to identify obstruction patterns to guide personalised treatment, especially in patients with continuous positive airway pressure (CPAP) therapy intolerance or complex OSA. The velopharynx has been identified as the region most susceptible to increased airflow resistance, negative pressure, collapse, and therefore obstruction site in OSA patients (Lambeth et al. 2018; Pirnar et al. 2015).

Effective treatment is essential to prevent severe health consequences. Lifestyle modifications such as weight loss, positional therapy, and avoidance of alcohol and sedatives are primary interventions (Jordan et al. 2014; Liistro et al. 1995). Weight‐loss interventions, including pharmaceutical agents such as tirzepatide (Malhotra et al. 2024), have shown promise in reducing AHI scores and improving OSA symptoms (Qaseem et al. 2013). Continuous positive airway pressure (CPAP) therapy remains the current gold standard in OSA management, with proven efficacy in improving AHI, oxygen saturation, daytime sleepiness, and sleep quality (Semelka et al. 2016; Qaseem et al. 2013). However, CPAP adherence is often hindered by challenges, such as mask discomfort and claustrophobia (Semelka et al. 2016; Peker et al. 2016; Sánchez‐de‐la‐Torre et al. 2020; Pépin et al. 2021).

Alternative treatment approaches include mandibular advancement devices (MADs) and other positive airway pressure modalities (e.g., bilevel positive airway pressure) (Qaseem et al. 2013). For patients with severe OSA or CPAP intolerance, surgical interventions, such as hypoglossal nerve stimulation (Kim et al. 2024), uvulopalatopharyngoplasty (UPPP), maxillomandibular advancement (MMA), or other upper airway surgeries have demonstrated significant efficacy (Aurora et al. 2010).

Especially MMA enlarges the whole UA and particularly the velopharyngeal region, the most frequent site of airway obstruction in OSA (Lee and Cho 2019). Consequently, airway collapsibility is reduced, and sustained improvements in AHI and symptom management (Boyd et al. 2019; Niskanen et al. 2019) are provided with this multilevel skeletal expansion. Several meta‐analyses conducted in the years 2018, 2021 and 2024 have demonstrated high surgical success rates above 80%, with curable outcomes as high as 46.3% cases (John et al. 2018; Zhou et al. 2021; Diemer et al. 2025).

The accurate identification and diagnosis of OSA is of significant importance as the previously mentioned medical consequences of an untreated condition are critical and potentially severe.

In OSA diagnostics, the AHI is the primary clinical parameter assessed during PSG. As all patients underwent cone beam computed tomography (CBCT) prior to MMA surgery, an extraction of additional information regarding the severity, localisation, and nature of the airway obstruction beyond what was available from PSG alone was made. These imaging datasets were then used for computational fluid dynamics (CFD) analysis. In 2020 an integrated and highly automated simulation‐based approach was introduced, using a CFD model to identify and localise anatomical causes of OSA (Pugachev et al. 2020). This method uses patient‐specific UA geometry and CFD simulation results to calculate the OSA index, a non‐dimensional parameter that reflects airway resistance. The study demonstrated a 91% accuracy in diagnosing OSA using the OSA index in a cohort of 63 patients with OSA and 17 controls (Pugachev et al. 2020), highlighting its potential as an alternative or adjunct criterion. The OSA index offers several advantages, including its ability to localise and quantify anatomical causes of airway obstruction and providing a more detailed understanding of OSA pathophysiology.

The objective of this study is to validate the PRI, a refinement of the original OSA index, as an additional diagnostic tool by comparison with established polysomnographic metrics, particularly the AHI, both before and after MMA surgery. The PRI is derived from CFD analysis based on standardised CBCT datasets performed with the software IPS inSilico × OSA from KLS Martin SE & Co. KG. Its purpose is to provide supplementary, patient‐specific insights into the anatomical and functional characteristics of upper airway obstruction. The current focus lies in further developing the PRI and assessing its clinical relevance by correlating it with AHI in a standardised cohort of 16 patients with OSA.

## 2 Methods

### 2.1 Cohort Characterisation

A retrospective, observational single‐center study was conducted based on a positive vote by the ethics committee of Charité—Universitätsmedizin Berlin (EA4/238/23).

Inclusion criteria consisted of a confirmed diagnosis of OSA in the patient's medical history, as well as the availability of standardised pre‐ and postoperative PSG reports and CBCT scans.

Exclusion criteria included non‐standardised PSG or CBCT data, an excessively long (> 24 months) or short (< 3 months) time interval between postoperative PSG and CBCT imaging, pregnancy, and absence of a diagnosed OSA.

Initially, 19 patients were evaluated. In two cases, OSA was not confirmed during follow‐up diagnostic workup, leading to their exclusion based on revised clinical classification. The third patient was excluded due to pregnancy, which introduces significant physiological changes in respiratory patterns and upper airway dynamics that may confound interpretation of both AHI and PRI measurements. Therefore, three patients were excluded from the final analysis due to the above‐mentioned exclusion criteria.

The 16 patients included received MMA surgery at the Department of Oral and Maxillofacial Surgery, Charité—Universitätsmedizin Berlin between November 2019 and June 2023.

All patients underwent a structured OSA diagnostic and treatment workflow, including comprehensive follow‐up. The diagnostic evaluation included PSG, CBCT imaging performed in accordance with a standardised protocol.

PSG data were obtained from multiple specialised sleep laboratories, each providing individual reports. Despite the variability in formatting across facilities, all reports exhibited a uniform incorporation of core standardised parameters according to current AASM criteria at the time of recording. These encompassed the respiratory frequency, number and type of respiratory events, sleep position, the AHI, the Respiratory Disturbance Index (RDI), and detailed information on sleep architecture, including the number of arousals, the durations of REM and deep sleep phases. To ensure data reliability, we only included reports explicitly stating adherence to AASM scoring rules and containing all core parameters. When multiple PSGs were available for a patient, the most recent report prior to surgery and the first follow‐up report after surgery were used. This approach minimised variability in scoring and ensured comparability across patients for the purposes of analysis.

The standardised imaging protocol for CBCT included standardised head positioning in sagittal direction according to the Frankfort Horizontal plane, in coronal direction aligned to the right and left orbital, and in central direction aligned to the Nasion. Patients were instructed to position their tongue against their front teeth and to refrain from breathing or swallowing during the scan. Prior to undergoing imaging procedures, pregnancy was ruled out in all finally included cases. CBCT scans were obtained using a KaVo 3D eXam system with the following parameters: field of view (FoV) 16 × 13 cm, voxel size 250 μm, 120 kV, 3.2 mA, and a scan time of 12.0 s.

Postoperative PSG and CBCT scans were achieved at least three to six months after surgery.

### 2.2 Descriptive Analysis

For descriptive analysis we included sex (male/female), age (in years), height (cm), weight (kg), BMI (kg/m^2^) and obesity classification (BMI > 25 kg/m^2^). We included preoperative and postoperative AHI and PRI values and deducted OSA classification.

### 2.3 Statistical Analysis

Statistical analysis was performed with R (Version 4.2.2, RStudio Team (2024), RStudio: Integrated Development for R, RStudio, PBC, Boston, MA). Data collection and diagram creation were performed using Microsoft Excel (version 16.78.3., Microsoft Corporation, Redmond, USA). Metric variables were checked on normal distribution with a Shapiro–Wilk test and then tested with Wilcoxon signed rank‐test. Additionally, the PRI values were tested with a paired *t*‐test. Pre‐ and postoperative class assignments of AHI and PRI were compared using the Wilcoxon signed‐rank test. Spearman's rank correlation was used to assess the association between AHI and PRI, as well as the correlation between their changes (ΔAHI vs. ΔPRI) to evaluate whether improvements in one parameter were associated with changes in the other. Significance thresholds were set at *p* < 0.05 (significant) and *p* < 0.01 (highly significant).

### 2.4 PRI and Its Origin

The PRI aims to identify OSA from CFD simulation results and incorporates geometric and fluid dynamic parameters. This is a further development of the OSA index presented in 2020 by Pugachev et al. (Pugachev et al. 2020). The simulation approach was experimentally validated using patient‐specific models (Pugachev et al. 2020; Arnold et al. 2021). The original OSA index was derived based on MRI data. The applicability of the PRI was extended to include CT and CBCT data. To facilitate better interpretation and easier comparison, the PRI was redefined to constrain its values within the range of 0–10.

CFD model allows detailed analysis of airflow dynamics and pressure changes in the UA, which are key to the pathophysiology of OSA. The PRI aggregates the most important outcomes of the CFD analysis into a dimensionless indicator developed to assess the anatomical causes of OSA and its severity. The PRI is defined as follows ($p_{i}$ are parameters, $p_{i}^{\mathrm{th}}$ are their threshold values, $w_{i}$ are weighting factors):

Formula 1: Definition of PRI.

$$\mathrm{PRI}\sim \sum_{i}\left(w_{i}\frac{p_{i}^{\mathrm{th}}-p_{i}}{\left|p_{i}^{\mathrm{th}}\right|}\right)$$

Explanation of Formula 1: The incorporated parameters (𝑝_𝑖_) describe the geometry of the pharynx and the air flow behaviour at each pharyngeal cross section during the breathing cycle. Threshold values (𝑝_𝑖_^𝑡ℎ^) and weighting factors (w_i_) were determined based on retrospective studies.

The set of parameters (and their respective threshold values/weighting factors) include the equivalent radius of the pharyngeal cross‐section, hydraulic resistance, area‐averaged air pressure, pressure gradient, air velocity, and shear strain rate. Based on the standardised effect size (Cohen's d), these parameters were identified as the most significant factors distinguishing the OSA patient group. An example of the PRI calculation is summarised in Table 1.

**TABLE 1:** Inputs used in the calculation and the resulting PRI for Patient 11.

	Equivalent radius, mm	Pressure, Pa	Pressure gradient, kg·m^−2^·s^−2^	Velocity, m/s	Shear Strain Rate, s^−1^	Hydraulic Resistance, N·s·m^−5^	PRI, −
Pre	4.58	−45.78	4823.10	8.56	4316.45	71212.57	1.73
Post	13.76	−3.00	19.06	0.96	343.50	3508.42	0.06

Abbreviation: PRI, pharyngeal resistance index.

The PRI results can be graded heuristically. Different PRI classes can be deducted, which can be seen in Table 2. The PRI classes are compared to AHI classes. PRI values close to 0 mean that the simulation results show no or insignificant indication of a sleep‐related breathing disorder. PRI values close to 10 mean that the simulation results show a strong indication of a severe sleep‐related breathing disorder.

**TABLE 2:** Heuristic grading of PRI compared to AHI in classes.

	AHI class	PRI class	OSA
1	< 5.0	< 0.1	No
2	5.0 … 15.0	0.1 … 1.0	Mild
3	15.0 … 30.0	1.0 … 6.0	Moderate
4	30.0 … 50.0	6.0 … 8.0	Severe
5	> 50.0	> 8.0	Very severe

Abbreviation: PRI, pharyngeal resistance index.

**FIGURE 1:**
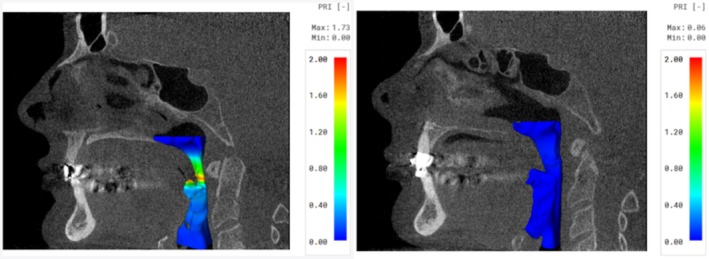
Visualisation of the PRI in 3D distribution for Patient 11 (preop—left, postop—right) (PRI, Pharyngeal Resistance Index).

The PRI can be visualised following 3D distribution along the pharynx at any given point of a breathing cycle. In Figure 1 the 3D distribution in CT‐imaging can be found pre‐ and postoperatively as an example for patient 11.

**FIGURE 2:**
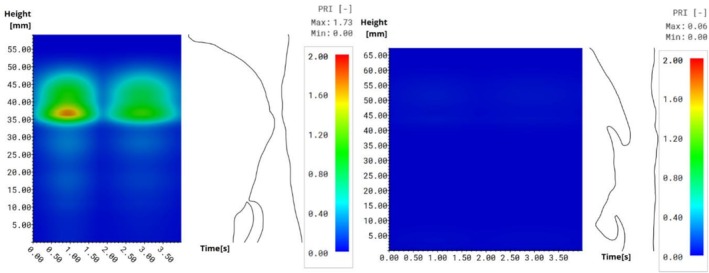
Visualisation of the PRI as a 2D pharyngeal map representing one breathing cycle for Patient 11 (preop—left, postop—right) (PRI, Pharyngeal Resistance Index).

Furthermore, the PRI can also be visualised as a 2D pharyngeal map representing the whole breathing cycle which can be seen in Figure 2. This 2D pharyngeal map is generated by dividing the pharynx into a number of transverse cross‐sections and calculating an averaged value at each cross‐sectional level and every time instant based on surrounding tissues. The horizontal axis represents time, and the vertical axis represents the long axis of the pharynx.

Please find the 3D distribution in CT‐imaging pre‐ and postoperatively as well as the 2D pharyngeal map of every patient in the Supporting Information.

## 3 Results

Initially, 19 patients were selected for our study. 16 patients (68.75% male (*n* = 11), 31.25% female (*n* = 5)) fulfilled inclusion criteria. The average age was 45.6 years (SD 9.71). The average height was calculated as 176.31 cm (SD 9.96), the average weight as 90.18 kg (SD 18.91) and consequently the mean BMI as 28.94 kg/m^2^ (SD 4.89). Following the WHO classification of obesity, two patients (12.5%, *n* = 16) were of normal weight (BMI 18.5–24.9 kg/m^2^), nine patients (56.25%, *n* = 16) classified as pre‐obese (BMI 25.0–29.9 kg/m^2^), four patients (25%, *n* = 16) presented with obesity class 1 (BMI 30.0–34.9 kg/m^2^), one patient (6.25%, *n* = 16) with obesity class 2 (BMI 35.0–39.9 kg/m^2^) and again one patient (6.25%, *n* = 16) with obesity class 3 (BMI > 40 kg/m^2^). Further information on the data described can be found in Table 3.

**TABLE 3:** Data overview of all selected patients.

#	Sex, Age	Height, cm	Weight, kg	BMI, kg/m^2^	AHI		PRI	
					Pre	Post	Pre	Post
1	F, 45	174	123	40.6	13.1	1.7	0.24	0.27
2	M, 53	172	83	28.1	2.9	2.5	1.51	0.02
3	F, 61	160	84	32.8	22.0	3.0	3.20	0.15
4	M, 54	165	93	34.2	27.0	11.8	1.08	0.29
5	M, 21	194	123	32.7	8.5	1.0	0.56	0.19
6	M, 51	196	130	33.8	79.1	17.2	0.70	0.07
7	M, 42	171	61.9	21.2	17.9	2.8	0.11	0.04
8	F, 44	172	83	28.1	5.4	2.1	0.63	0.08
9	M, 39	179	80	25.0	43.3	2.9	0.37	0.09
10	M, 43	170	86	29.8	9.0	1.3	0.41	0.05
11	F, 57	173	75	25.1	21.2	5.0	1.73	0.06
12	M, 35	179	85	26.5	5.1	1.6	0.68	0.04
13	M, 37	185	78	22.8	11.0	2.0	0.78	0.01
14	M, 49	182	90	27.2	16.4	0.6	1.40	0.69
15	F, 49	166	78	28.3	22.0	0.8	0.20	0.02
16	M, 49	183	90	26.9	70.8	12.8	1.82	0.67
17	M, 30	184	90	26.6	6.6		0.18	
18	F, 40	168	66.5	23.6	3.0		0.99	
19	F, 53	174	79	26.1	11.1		0.27	

*Note:* Grey‐shaded patients were not included.

Abbreviation: PRI, pharyngeal resistance index.

**FIGURE 3:**
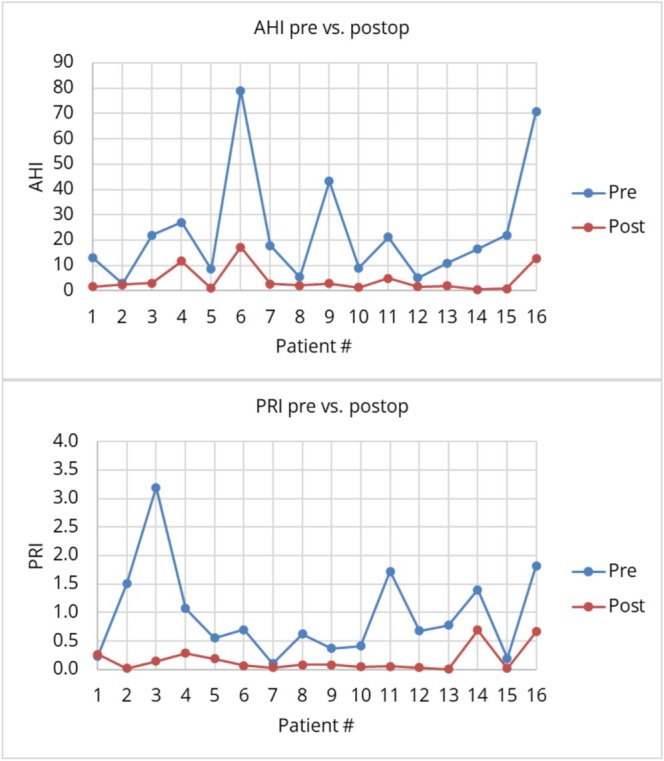
AHI and PRI pre vs. post MMA surgery (AHI, Apnea‐hypopnea index; MMA, maxillomandibular advancement; Patient #, Patient number; PRI, pharyngeal resistance index).

In Figure 3 the reduction of AHI and PRI after MMA surgery can be found.

**FIGURE 4:**
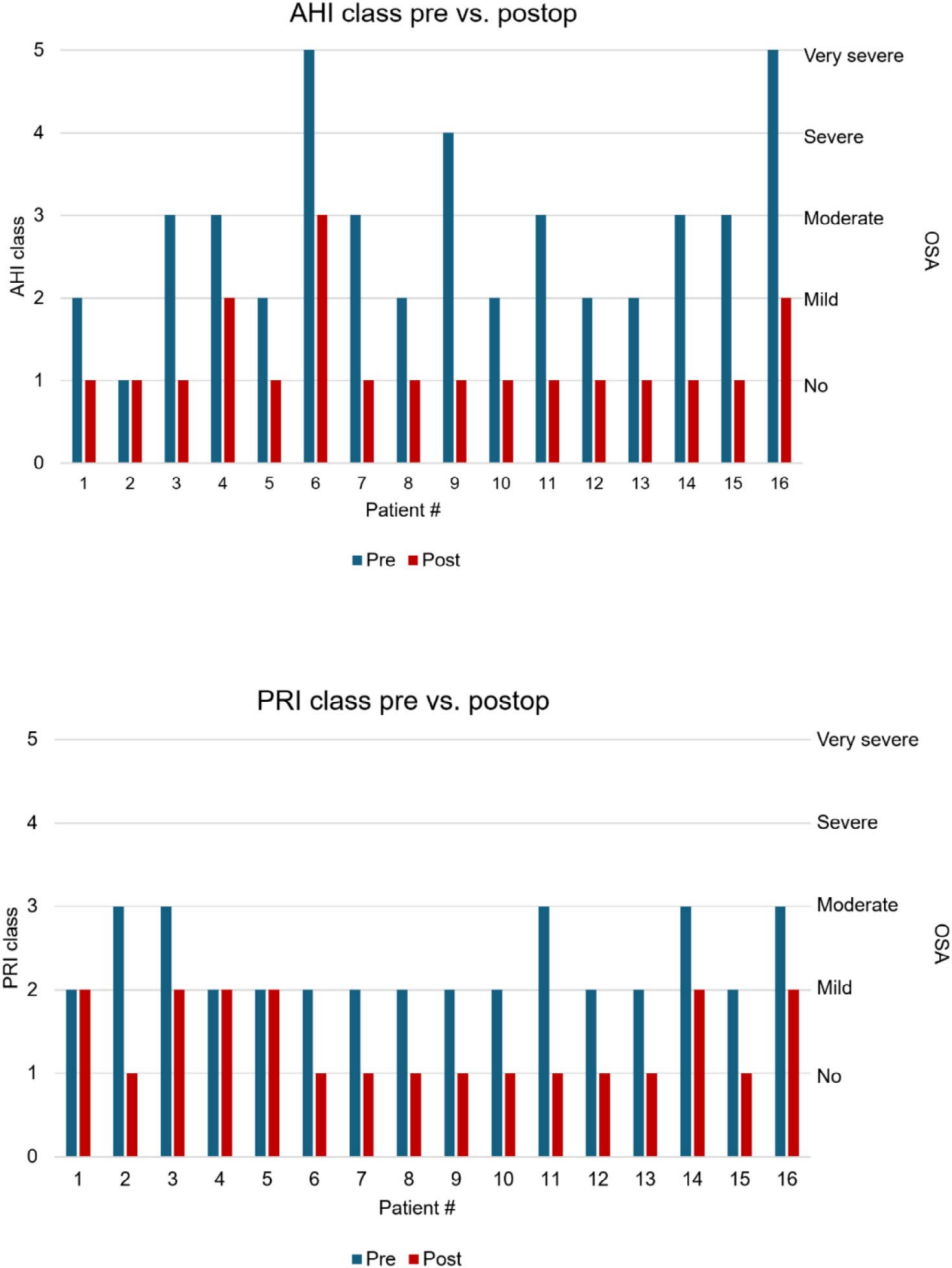
Improvement of AHI and PRI class. Comparison of AHI and PRI class pre vs. post MMA surgery (AHI, Apnea‐hypopnea index; MMA, maxillomandibular advancement; Patient #, Patient number; PRI, pharyngeal resistance index).

The AHI and PRI values can be further categorised into OSA severity classes and are shown in Figure 4 (*n* = 16). Based on preoperative AHI values, one patient (6.25%) was diagnosed with no OSA six patients (37.5%) had mild or moderate OSA, one patient (6.25%) with severe OSA, and 2 patients (12.5%) with severe OSA. After MMA surgery, the distribution increased to 13 patients (81.25%) with no OSA, two patients (12.5%) with mild OSA, and one patient (6.25%) moderate OSA. Clearly a shift toward lower OSA severity can be recognised (Wilcoxon signed‐rank test, *p* < 0.001). Higher severity was virtually eliminated after MMA surgery.

Regarding preoperative PRI values, eleven patients (68.75%) were classified as mild OSA and five patients (31.25%) as moderate OSA with no cases of no, severe or very severe OSA. Postoperatively a similar trend as in the AHI values was observed—ten patients (62.5%) were classified as no OSA, and six patients (37.5%) as mild OSA with no patients falling into higher severity categories. This shift was also statistically significant (Wilcoxon signed‐rank test, *p* < 0.001).

**FIGURE 5:**
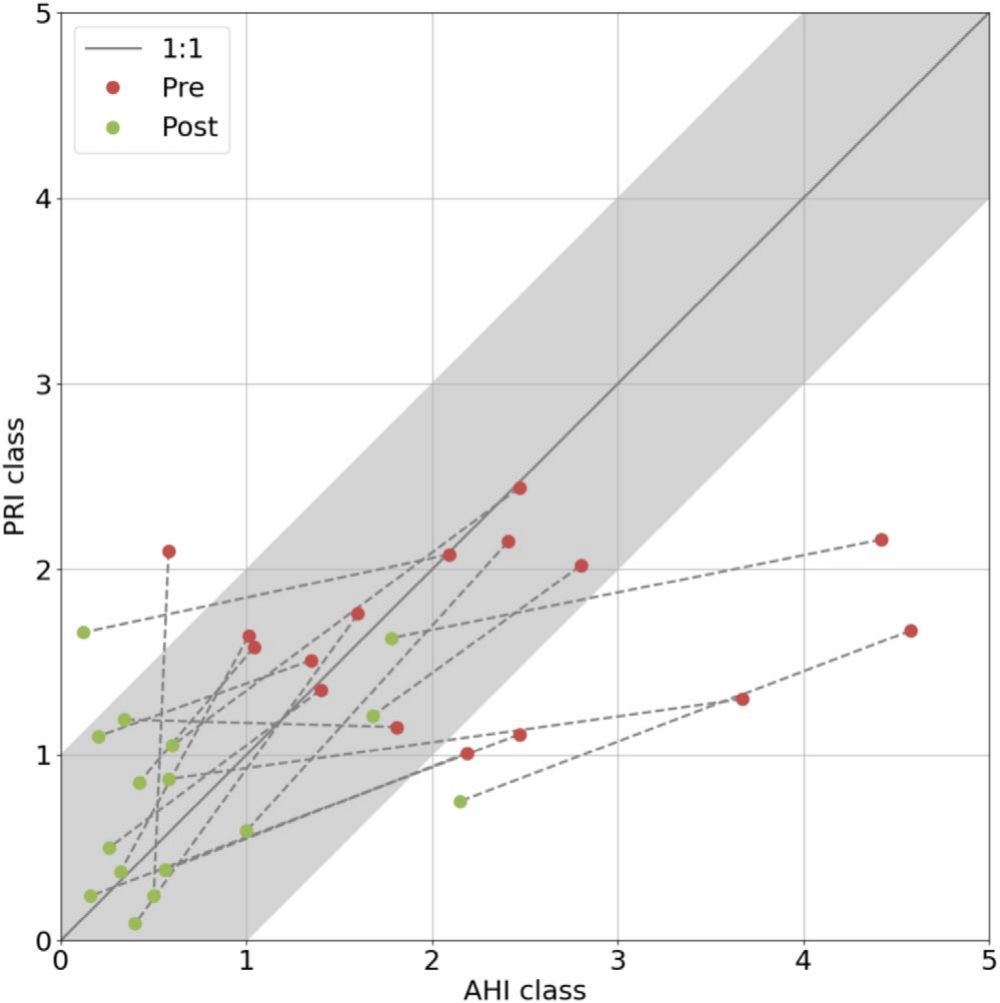
Scatterplot comparing the pre‐ and postoperative AHI and PRI class assignments. (AHI, Apnea‐hypopnea index; PRI, pharyngeal resistance index).

The AHI and PRI class assignments were plotted both pre‐ and postoperatively (Figure 5). The red dots represent the preoperative values; the green dots represent the postoperative values. Each pair of dots corresponds to the same patient measured before and after MMA surgery. The grey diagonal line indicates perfect class agreement (AHI class = PRI class). The shaded band denotes an acceptable range of ±1 class deviation. Preoperatively, a systematic discrepancy was identified: PRI exhibited a tendency to assign patients to lower severity classes than AHI, resulting in red dots with a high AHI value to fall below the line of identity (1:1 diagonal). Several patients showed differences exceeding the boundaries of one class, thereby indicating that PRI may underestimate the severity of OSA in the pre‐treatment state. This discrepancy reflects the different physiological emphasis of the PRI compared to AHI.

Postoperatively, there was a significant improvement in AHI and PRI class agreement. Most green dots clustered in the lower left quadrant and therefore within ±1 class difference, indicating a clinically adequate level of agreement between AHI and PRI following intervention. The downward trend of the dashed lines from red (pre) to green (post) also highlights the reduction in PRI and AHI class. This signifies mild or no residual disease, as indicated by both indices.

A Shapiro–Wilk test was performed to analyse the distribution of pre‐ and postoperative AHI and PRI values. The Shapiro–Wilk test revealed that none of the AHI or PRI values (pre‐ and postoperative) were normally distributed (*p* < 0.05, *n* = 16). Based on these findings, a Wilcoxon signed‐rank test was conducted to assess the mean pre‐ and postoperative AHI and PRI values.

The median preoperative AHI was 17.15 (95% CI: 9.0–24.1) and decreased to 2.30 (95% CI: 1.60–3.75) postoperatively. This represents a mean reduction of 81.55% (*n* = 16) (*p* < 0.01), indicating a highly significant difference between pre‐ and post‐MMA surgery AHI values. The median preoperative PRI decreased from 0.69 (95% CI: 0.41–1.40) to 0.08 (95% CI: 0.04–0.19) postoperatively. This represents a mean reduction of 82.29% (*n* = 16) (*p* < 0.01), thereby suggesting a highly significant improvement in PRI values following MMA surgery.

Spearman rank correlation was calculated to assess both indices' relationship. No significant correlation was observed between AHI and PRI values either preoperatively (ρ = 0.130, *p* = 0.633) or postoperatively (ρ = 0.159, *p* = 0.556). In addition, the correlation between the changes in AHI (ΔAHI) and PRI (ΔPRI) was assessed using Spearman's rank correlation. No significant association was found (ρ = 0.059, *p* = 0.829), indicating that the magnitude of improvement in AHI and PRI did not systematically covary across patients.

## 4 Discussion

This study examines the AHI and the PRI both before and after MMA surgery, highlighting the diagnostic value in the context of OSA evaluation. The AHI serves as the primary clinical parameter for diagnosing and assessing the severity of OSA. However, it has limitations, particularly in accurately localising and identifying the origin and nature of airway obstructions. In order to overcome these limitations, 3D imaging was implemented to perform CFD analyses. These analyses incorporated both static parameters (such as the minimal cross‐sectional area (MCSA) and anatomical landmarks) and dynamic parameters (including airflow velocity and pressure distribution). From this, a novel index—the PRI—was developed and subsequently compared to changes in AHI. Due to its high sensitivity to anatomical and functional changes, the PRI has the potential to become a valuable diagnostic tool in future OSA evaluation and may contribute to predicting the success of individualised treatment strategies.

### 4.1 AHI and Its Limitations

The AHI has traditionally served as the primary clinical parameter for diagnosing OSA, but it has limitations. AHI assessment requires patients to undergo overnight polysomnography in a specialised facility, which can be both inconvenient and expensive (Qaseem et al. 2013; Ryu et al. 2021). In addition, limited availability of sleep lab appointments often leads to long waiting times, delaying timely diagnosis and treatment, and potentially increasing long‐term healthcare costs due to the progression of untreated OSA (Kapur et al. 2017; Phua et al. 2021; Natsky et al. 2022). Furthermore, the AHI shows internight variability, influenced by factors such as sleep position or sleep stage distribution. This variability can lead to inconsistent severity classification and treatment decisions based on single‐night attended polysomnography (Martinot et al. 2023). As previously stated, the AHI also falls short in accurately localising and identifying the origin and nature of airway obstructions.

To address these shortcomings, further research has introduced additional hypoxia indices (Hui et al. 2024). These indices focus on characterising OSA severity with its complications, particularly those related to cardiovascular health. For example, a new parameter called Hypoxic Burden (HB) measures the cumulative oxygen desaturation associated with OSA during sleep (Azarbarzin et al. 2019), allowing a predictive assessment for cardiovascular disease mortality risk. Similarly, the sleep breathing impairment index (SBII) incorporates the frequency, duration, and associated oxygen desaturation levels of respiratory events and has been shown to correlate with the risk of cardiovascular morbidity in OSA patients (Cao et al. 2023).

In addition to hypoxic indices, structural and functional aspects of the UA have been increasingly emphasised in the diagnosis and management of OSA. Imaging modalities play a crucial role in this context. For instance, three‐dimensional craniofacial and UA anatomical variables were assessed by CBCT to show a correlation with the AHI and, consequently, the severity of OSA (Wang et al. 2021). Advanced techniques, including cine MRI or DISE, provide detailed visualisation of the level, extent, and type of obstruction in OSA patients (Garg et al. 2024; Ulualp and Kezirian 2024). These techniques facilitate the prediction of structural and dynamic factors contributing to airway obstruction but still are not without limitations. Cine MRI, for example, offers dynamic imaging capabilities but remains costly and is limited in availability, making widespread clinical application challenging (Ulualp and Kezirian 2024; Volner et al. 2022). DISE represents another diagnostic approach, providing direct visualisation of airway collapse during pharmacologically induced sleep. However, DISE requires anaesthesia, poses additional risks to the patient, is not universally covered by public health insurance, and may delay timely therapeutic intervention due to logistical and financial barriers (Vroegop et al. 2020; Kotecha and De Vito 2018).

Still DISE provides dynamic assessment of airway behaviour during sleep, offering an advantage over static CBCT imaging obtained while awake. CBCT cannot capture sleep‐related airway collapsibility, as muscle tone is higher during wakefulness and decreases in deeper sleep stages, particularly REM, leading to greater airway obstruction. Although OSA patients show smaller upper airway dimensions on CBCT compared with controls, these measurements may underestimate the true degree of sleep‐related narrowing (Gurgel et al. 2023). Consequently, awake imaging does not fully represent the pathophysiology of sleep‐disordered breathing. CFD model analysis has emerged as a promising diagnostic tool, providing detailed insights into airflow dynamics and pressure changes within the UA by using imaging data (e.g., CT or MRI). Specific key determinants of airway collapsibility including the MCSA and pharyngeal critical closing pressure (Pcrit) can be assessed. For example, a CFD model analysis has demonstrated diagnostic value by analysing UA effective compliance (the slope of MCSA vs. pressure) which correlated with Pcrit and the AHI and provided a biomechanical perspective of the UA during wakefulness and sleep (Choy et al. 2021). Another significant advantage of CFD analysis is that imaging data can be obtained during wakefulness, potentially reducing reliance on attended polysomnography.

Conclusively, CFD modelling has the potential to provide more precise anatomical and functional identification of obstructive sites compared to attended polysomnography and the AHI (Powell et al. 2011). By predicting and analysing airflow characteristics based on anatomical morphology extracted from imaging modalities, CFD models enable highly accurate diagnoses, particularly for moderate OSA (Ryu et al. 2021; Wootton et al. 2014). This precision facilitates the development of individualised treatment strategies, such as surgical intervention.

Surgical approaches, like MMA or UPPP, have been shown to be effective in improving AHI postoperatively, as confirmed by several studies using CFD models (Furundarena‐Padrones et al. 2024; Chang et al. 2018). For example, Nomura et al. combined CT imaging with CFD analysis to evaluate airflow in the UA before and after UPPP and successfully predict postoperative function (Nomura et al. 2022). Pugachev et al. used CFD modelling to predict improved outcomes with a MAD device treatment (Pugachev et al. 2020).

### 4.2 PRI With Its Advantages and Disadvantages

The ability of the PRI to provide both two‐ and three‐dimensional visualisations of the UA and pharynx further facilitates decision‐making for individualised treatment strategies. By integrating those advanced tools, patients could benefit from more accurate diagnosis and tailored interventions, ultimately improving clinical outcomes. Please find a detailed table showing the localisation and origin of airway obstruction of all 16 patients in the Supporting Information.

The PRI incorporates additional physiological features including airflow dynamics and pressure changes in the UA, which are crucial in order to understand the pathophysiology of OSA. Kazemeini et al. describe five key pathophysiological factors affecting the UA and ventilation during sleep: site and pattern of the collapse in the UA, collapsibility of the UA, ventilatory control stability, pharyngeal muscle responsiveness, and arousal threshold (Kazemeini et al. 2022).

As a calculated index, the PRI is generated from CFD model analysis by dividing the pharynx into a number of transverse cross‐sections. At each pharyngeal cross‐sectional level, values were calculated on average for selected geometric and fluid dynamic parameters and every time instant based on surrounding tissues (Pugachev et al. 2020). These include the equivalent radius of the pharyngeal cross‐section, hydraulic resistance, area‐averaged air pressure, pressure gradient, air velocity, shear strain rate, threshold values, and weighting factors from CT and CBCT data. Therefore, the PRI incorporates multiple aspects of pathophysiological key factors and allows highly individual assessment of airway obstruction and OSA severity.

The PRI is intended as a complementary diagnostic metric rather than a direct substitute for the AHI. Notably, Spearman's rank correlation analysis revealed no significant correlation between the PRI and the AHI, both preoperatively (ρ = 0.130, *p* = 0.633) and postoperatively (ρ = 0.159, *p* = 0.556) in our cohort. Furthermore, no significant correlation was observed between the changes in AHI (ΔAHI) and PRI (ΔPRI) (ρ = 0.059, *p* = 0.829), indicating that the magnitude of improvement in one index does not necessarily predict improvement in the other. This finding highlights that AHI and PRI capture distinct but complementary aspects of upper airway obstruction. The AHI focuses on discrete respiratory events counts, while the PRI incorporates additional physiological dimensions such as airflow dynamics and pressure changes in the UA. By integrating these parameters, the PRI addresses limitations of the AHI by incorporating measures beyond event frequency. The visual distribution of pre‐ and postoperative class assignments (Figure 5) complements the correlation analysis between the AHI and the PRI: preoperatively, the deviation from the 1:1 line emphasises how PRI classifies patients differently, often implying potential underestimation of disease burden. This finding is consistent with the low Spearman correlation coefficient observed on the preoperative site (ρ = 0.130, *p* = 0.633).

Postoperatively, however, a shift is evident. The majority of postoperative data points fell around the 1:1 line within ±1 class deviation. This pattern reflects a substantial alignment between AHI and PRI classifications and indicates a clinically acceptable level of agreement following intervention. Importantly, although individual discrepancies persist, they are confined to a single class. This observation is in line with the slightly increased, though still nonsignificant, correlation in the postoperative setting (ρ = 0.159, *p* = 0.556). With both indices reflecting therapeutic improvement, a convergence in clinical trajectory is indicated.

Although PRI and AHI do not rank patients identically, this divergence reflects their differing emphases rather than inconsistency. The PRI may be particularly sensitive to changes in disease state that are not fully reflected by frequency‐based metrics alone, making it a clinically meaningful and complementary diagnostic tool. Especially in the context of surgical interventions, where established indices like AHI may underestimate residual burden or fail to reflect qualitative improvements, the PRI emerges as a promising feature for more nuanced and multidimensional monitoring of treatment outcomes. As a digitally derived metric, the PRI aligns with the broader trend of innovation in orthognathic surgery, alongside emerging tools such as dental MRI. Although CBCT continues to be the prevailing standard for virtual surgical planning in orthognathic surgery, dental MRI, with its capacity for detailed soft tissue visualisation, demonstrates considerable promise as an alternative to conventional radiography. In the future, the integration of these evolving technological advancements, particularly the PRI and dental MRI, into virtual surgical planning for orthognathic surgery may contribute substantially to enhanced individualised treatment strategies, enabling customised surgical approaches, improved predictability, precise occlusal analysis and simulation, reduced surgical complications and shorter operative times (Kobravi et al. 2025; Flügge et al. 2023).

### 4.3 Limitations

1. The present study is not without limitations. First, the temporal discrepancy between the administration of PSG and CBCT imaging may introduce variability and limit the direct comparability of results. Second, data were obtained from multiple sleep laboratories, leading to heterogeneity in how respiratory disturbances were defined and reported, which may have affected data consistency across the sample. Third, the study faltered to differentiate between desaturation‐correlated and “normal” as well as central and obstructive respiratory events. This methodological limitation may have influenced the analysis and interpretation of the data. Additionally, the study did not account for the differences in respiratory event frequencies depending on sleep position, particularly between total events and those in the supine position.
2. The presented PRI‐based assessment method offers a practical approach to evaluating OSA, but it is also not without limitations. Radiological data were acquired during wakefulness and may not accurately reflect dynamic upper airway geometry during sleep. Sleep related changes in PRI may therefore show a different correlation with AHI than measurements obtained awake. Although a standardised CBCT scan protocol was applied, patient compliance remains critical. Movements such as breathing, swallowing, or tongue repositioning can alter the static pharyngeal volume captured by CBCT, potentially affecting measurement accuracy. Moreover, the conventional requirement of specialised software and expertise in conducting CFD analysis serves as a barrier to its clinical implementation (Qaseem et al. 2013; Xiao et al. 2024). To overcome these challenges, the workflow was fully automated with IPS inSilico × OSA, and the PRI was introduced as a balanced compromise—a user‐friendly, easily interpretable indicator suitable for clinical application through necessary simplifications while still providing meaningful anatomical and functional insights into upper airway obstruction. The findings of this study suggest that, with appropriate parameter calibration, the PRI is a reliable and accessible tool for the assessment of obstructive sleep apnea.
3. These limitations should be considered when interpreting the study's results and their generalizability.

## 5 Conclusion

This study sheds light on the potential of the PRI for the diagnosis and treatment planning in patients with OSA. Although the PRI conducted from CFD‐model analysis cannot replace the gold standard of AHI conducted from PSG, it offers complementary anatomical and functional insights in patients with OSA and may ultimately facilitate individualised treatment planning. Despite the promising results, further clinical data need to be analysed to validate the developed index before it can be used and recommended in a real‐world clinical setting.

## Author Contributions

Conceptualization: S.B., T.E., and A.P. Data curation: N.L.K., A.P., and S.B. Formal analysis: A.P. and N.L.K. Funding acquisition: T.E., M.H., and S.B. Investigation: A.P. and N.L.K. Methodology: A.P. and S.B. Project administration: S.B. Resources: A.P., M.H., S.B., and A.B. Software: A.P. Supervision: S.B. Validation: All authors. Visualization: N.L.K., A.P., and S.B. Writing – original draft preparation: N.L.K. Writing – review and editing: All authors.

## Funding

This study was supported by KLS Martin Group. Additionally, KLS Martin provides support to Simq GmbH.

## Ethics Statement

Approval was given by the ethics committee of Charité—Universitätsmedizin Berlin (EA4/238/23).

## Consent

Patient consent was waived due to the retrospective design of the study and the exclusive use of anonymised data, in accordance with approval by the institutional ethics committee.

## Conflicts of Interest

T.E. has received compensation for travel costs from DePuy Synthes, and speaker's fees from K.L.S. Martin, M.H. has received compensation for travel costs, speaker's fees, and research funds from K.L.S. Martin, and speaker's fees from Materialise. A.P., T.L.E., N.L.K., A.B., J.O.V., and S.B.: No conflicts of interest.

## Supporting information


**Data S1:** Supporting Information.

## Data Availability

The data that support the findings of this study are available from the corresponding author upon reasonable request.
