# Supplementary material for: The Pharyngeal Resistance Index—A Promising Diagnostic Tool in OSA
Source: J Sleep Res. 2026 Apr 13;35(4):e70341. doi: 10.1111/jsr.70341 (PMC13357853; doi:10.1111/jsr.70341)
Supplement: Supplementary file 1 — Data S1: Supporting Information. [file JSR-35-e70341-s001.docx]

| **Patient number (#)** | **Sex/Age** | **Height (cm)/Weight (kg)/BMI (kg/m^2^)** | **AHI pre / post** | **PRI pre / post** | **AHI class pre / post** | **PRI class pre / post** | **CBCT/PSG pre** | **CBCT/PSG post** | **Further**  **Follow**  **Ups** |
| --- | --- | --- | --- | --- | --- | --- | --- | --- | --- |
| 1 | F / 45 | 174 / 123 / 40.6 | 13.10 / 1.70 | 0.24 / 0.27 | 1.8 / 0.3 | 1.2 / 1.2 | 25.06.2020 | 29.03.2022 |  |
| 2 | M / 53 | 172 / 83 / 28.1 | 2.90 / 2.50 | 1.51 / 0.02 | 0.6 / 0.5 | 2.1 / 0.2 | 04.02.2021 | 21.09.2021 |  |
| 3 | F / 61 | 160 / 84 / 32.8 | 22.00 / 3.00 | 3.20 / 0.15 | 2.5 / 0.6 | 2.4 / 1.1 | 29.04.2019 | 20.11.2019 |  |
| 4 | M / 54 | 165 / 93 / 34.2 | 27.00 / 11.80 | 1.08 / 0.29 | 2.8 / 1.7 | 2.0 / 1.2 | 26.10.2022 | 16.03.2023 |  |
| 5 | M / 21 | 194 / 123 / 32.7 | 8.50 / 1.00 | 0.56 / 0.19 | 1.4 / 0.2 | 1.5 / 1.1 | 19.10.2020 | 11.01.2023 |  |
| 6 | M / 51 | 196 / 130 / 33.8 | 79.10 / 17.20 | 0.70 / 0.07 | 4.6 / 2.1 | 1.7 / 0.7 | 15.02.2022 | 31.08.2022 |  |
| 7 | M / 42 | 171 / 61.9 / 21.2 | 17.90 / 2.80 | 0.11 / 0.04 | 2.2 / 0.6 | 1.0 / 0.4 | 07.10.2021 | 31.05.2022 |  |
| 8 | F / 44 | 172 / 83 / 28.1 | 5.40 / 2.10 | 0.63 / 0.08 | 1.0 / 0.4 | 1.6 / 0.8 | 27.09.2021 | 08.11.2022 | 22.11.2022 |
| 9 | M / 39 | 179 / 80 / 25.0 | 43.30 / 2.90 | 0.37 / 0.09 | 3.7 / 0.6 | 1.3 / 0.9 | 07.12.2021 | 03.11.2022 |  |
| 10 | M / 43 | 170 / 86 / 29.8 | 9.00 / 1.30 | 0.41 / 0.05 | 1.4 / 0.3 | 1.3 / 0.5 | 22.07.2021 | 20.06.2023 |  |
| 11 | F / 57 | 173 / 75 / 25.1 | 21.20 / 5.00 | 1.73 / 0.06 | 2.4 / 1.0 | 2.1 / 0.6 | 07.09.2020 | 01.11.2021 |  |
| 12 | M / 35 | 179 / 85 / 26.5 | 5.10 / 1.60 | 0.68 / 0.04 | 1.0 / 0.3 | 1.6 / 0.4 | 21.03.2022 | 13.10.2022 |  |
| 13 | M / 37 | 185 / 78 / 22.8 | 11.00 / 2.00 | 0.78 / 0.01 | 1.6 / 0.4 | 1.8 / 0.1 | 02.11.2021 | 31.10.2022 |  |
| 14 | M / 49 | 182 / 90 / 27.2 | 16.40 / 0.60 | 1.40 / 0.69 | 2.1 / 0.1 | 2.1 / 1.7 | 10.04.2019 | 11.05.2020 | 06.10.2020/  26.05.2021 |
| 15 | F / 49 | 166 / 78 / 28.3 | 22.00 / 0.80 | 0.20 / 0.02 | 2.5 / 0.2 | 1.1 / 0.2 | 08.11.2021 | 13.09.2022 |  |
| 16 | M / 49 | 183 / 90 / 26.9 | 70.80 /12.80 | 1.82 / 0.67 | 4.4 / 1.8 | 2.2 / 1.6 | 28.06.2021 | 02.02.2023 |  |
| 17 | M / 30 | 184 / 90 / 26.6 | 6.60 / - | 0.18 / - | 1.2 / - | 1.1 / - |  |  |  |
| 18 | F / 40 | 168 / 66.5 / 23.6 | 3.00 / - | 0.99 / - | 0.6 / - | 2.0 / - |  |  |  |
| 19 | F / 34 | 174 / 79 / 26.1 | 11.10 / - | 0.27 / - | 1.6 / - | 1.2 / - |  |  |  |

Supplementary materials

Caption Table S3: Patients’ characteristics. Grey-shaded patients were not included. (AHI=apnea-hypopnea index; PRI= Pharyngeal Resistance Index; CBCT=cone beam computed tomography; PSG=polysomnography)

Caption Figure S6: Localization and origin of airway obstruction of all 16 patients: pre- and postoperative 3D distribution in CT-imaging and 2D pharyngeal maps showing the PRI (PRI= Pharyngeal Resistance Index).

| Patient | Pre | Post |
| --- | --- | --- |
| 1 | 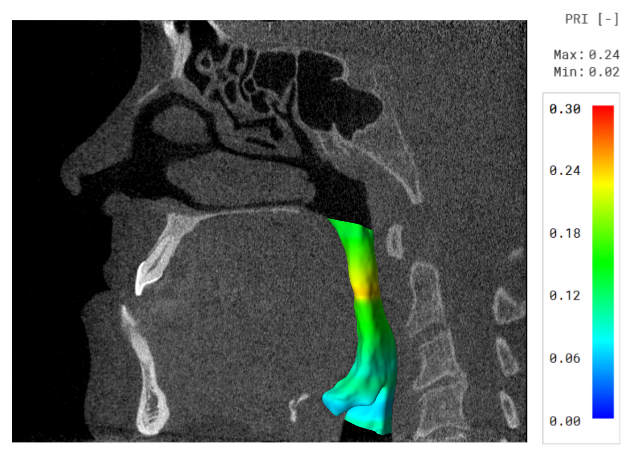 | 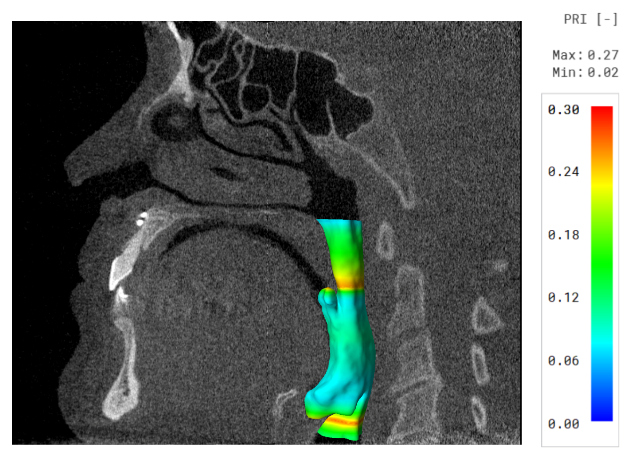 |
|  | 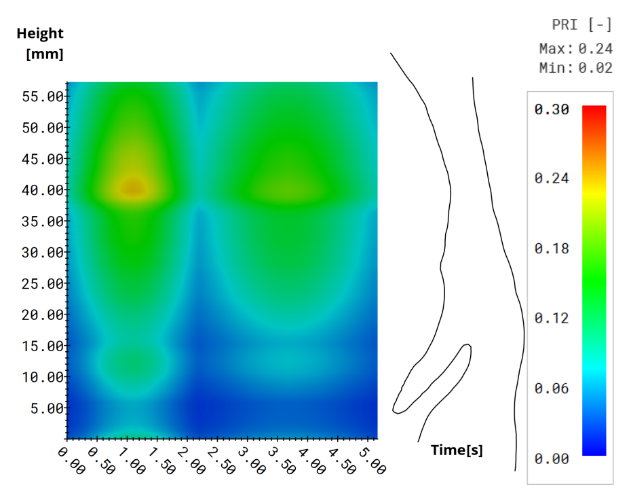 | 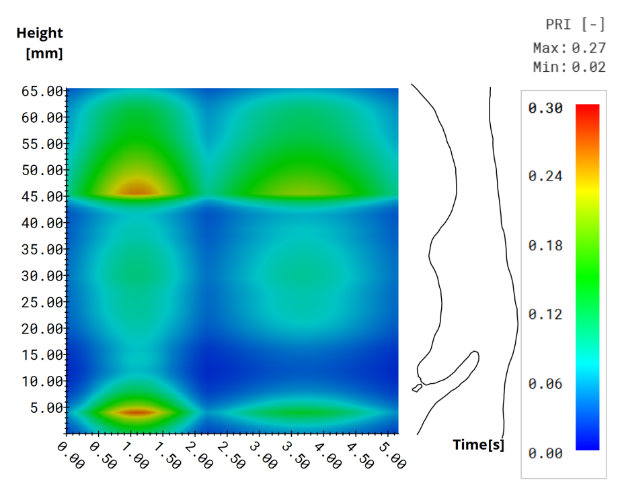 |
| 2 | 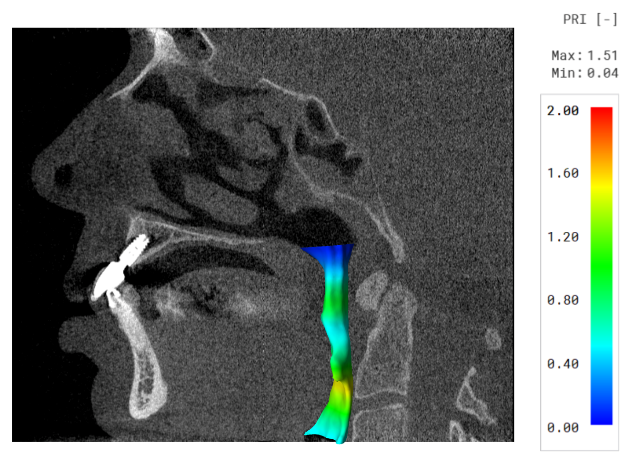 | 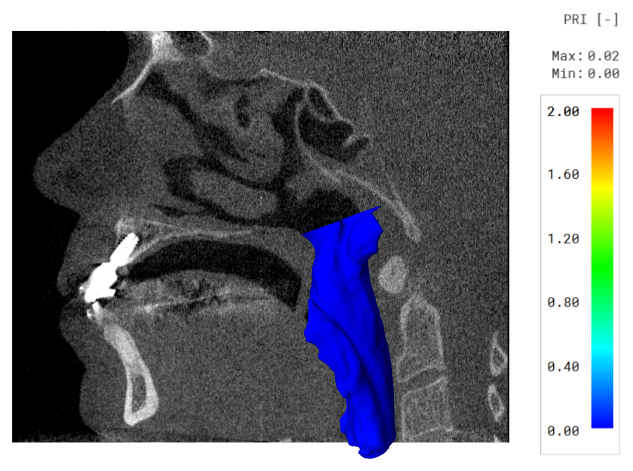 |
|  | 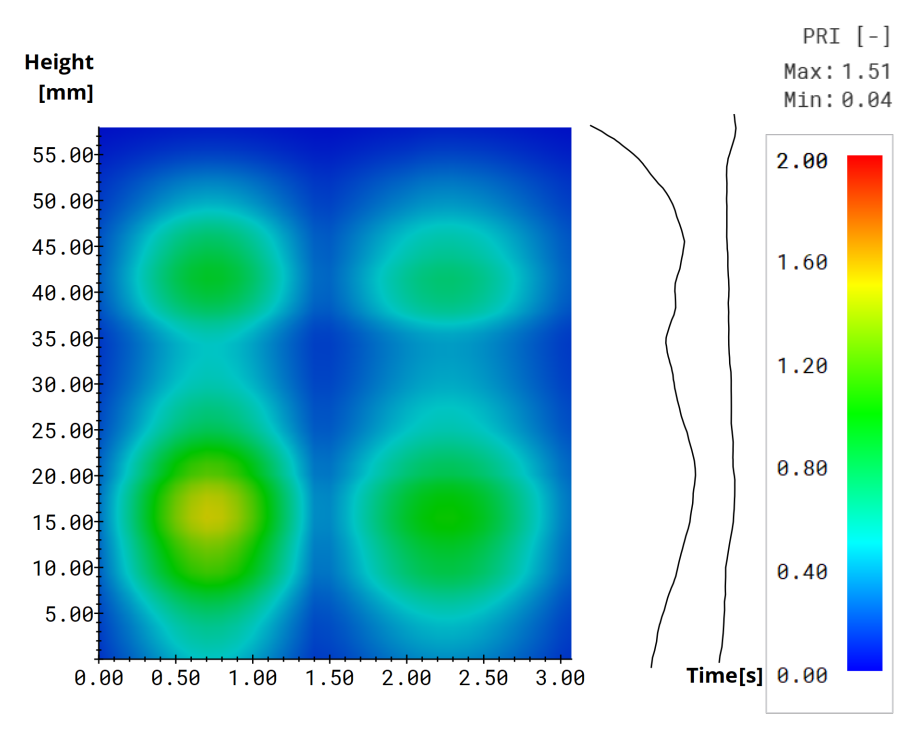 | 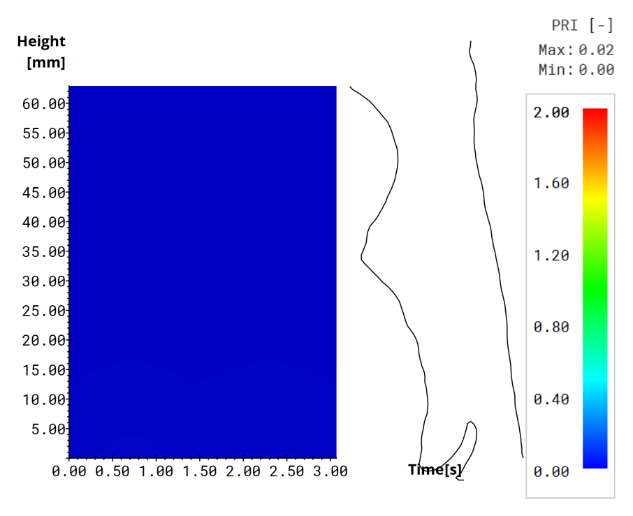 |
| 3 | 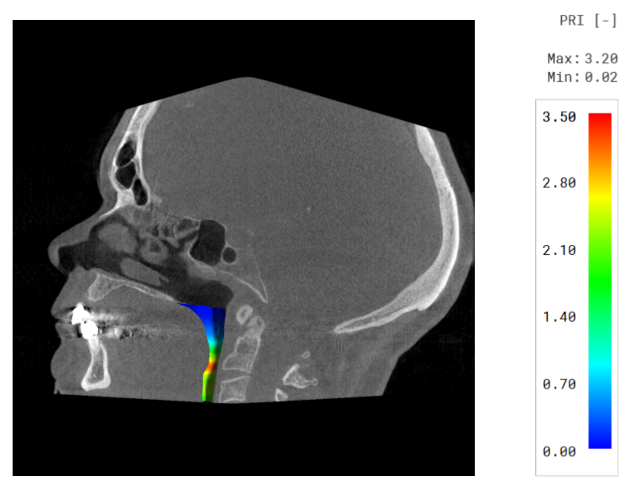 | 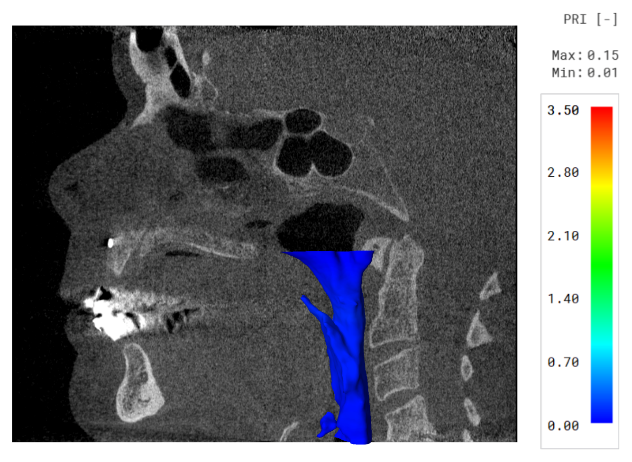 |
|  | 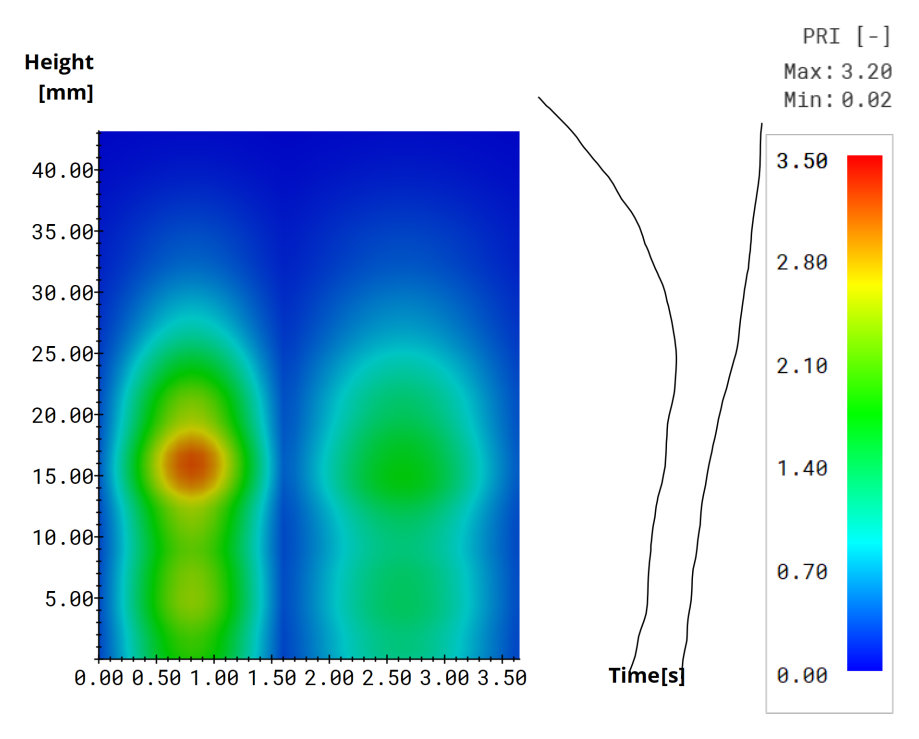 | 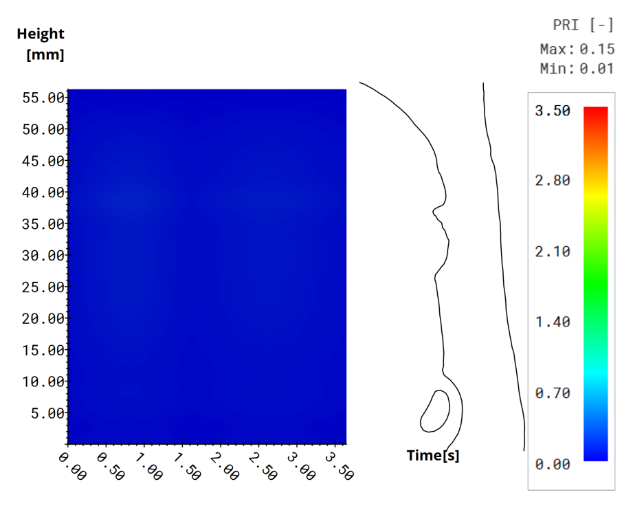 |
| 4 | 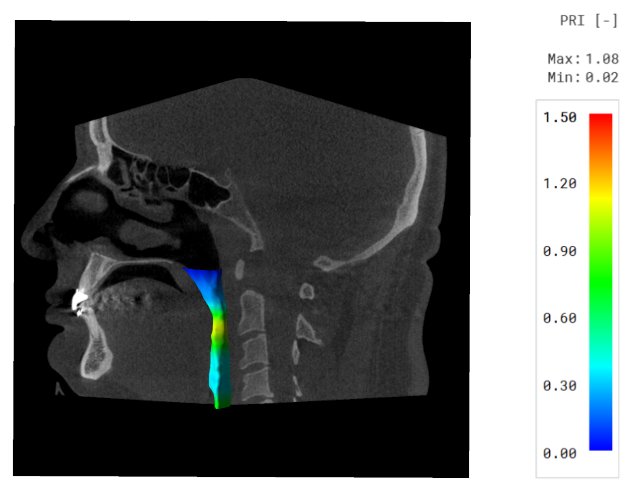 | 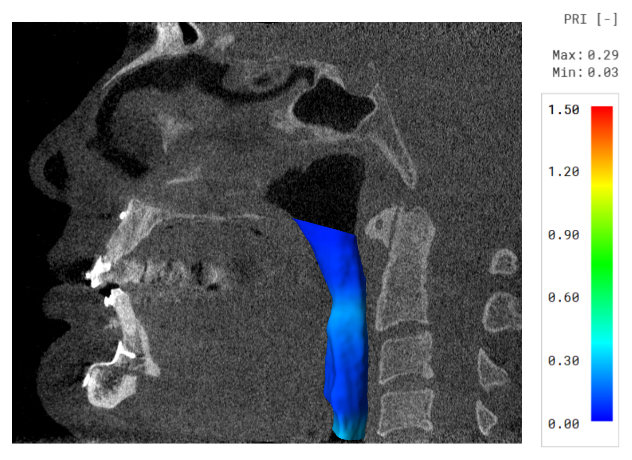 |
|  | 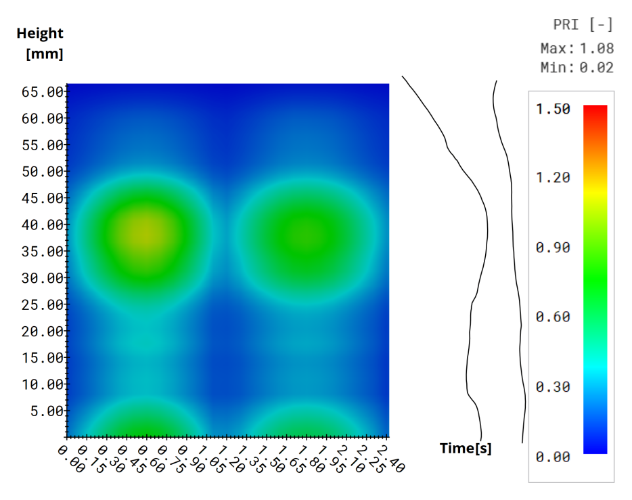 | 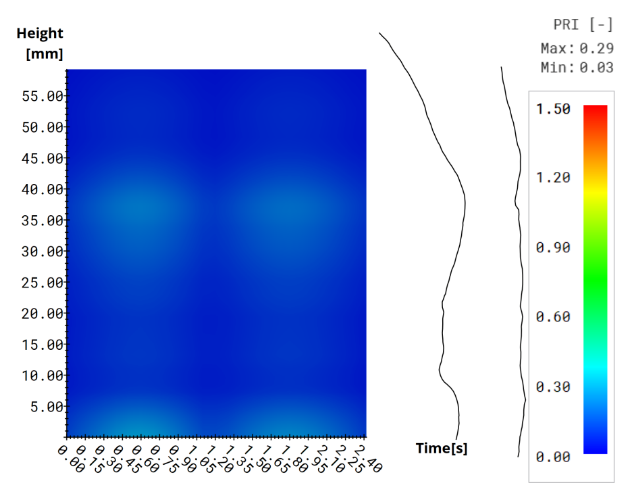 |
| 5 | 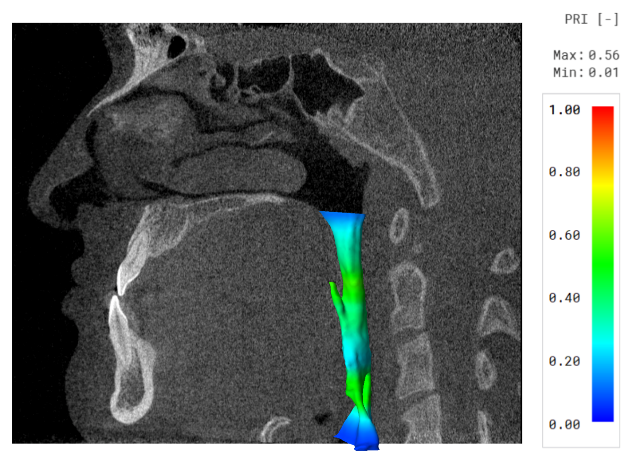 | 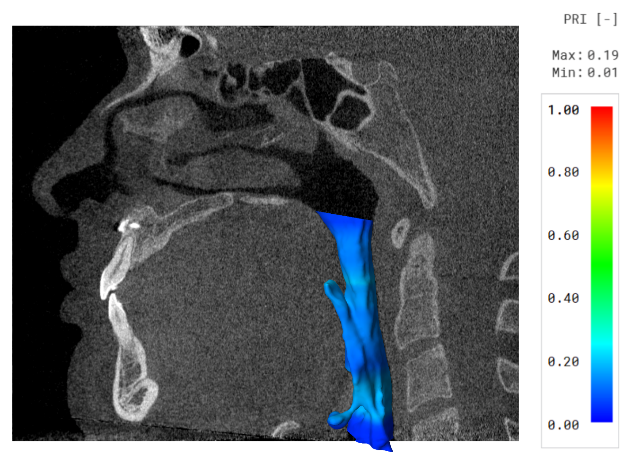 |
|  | 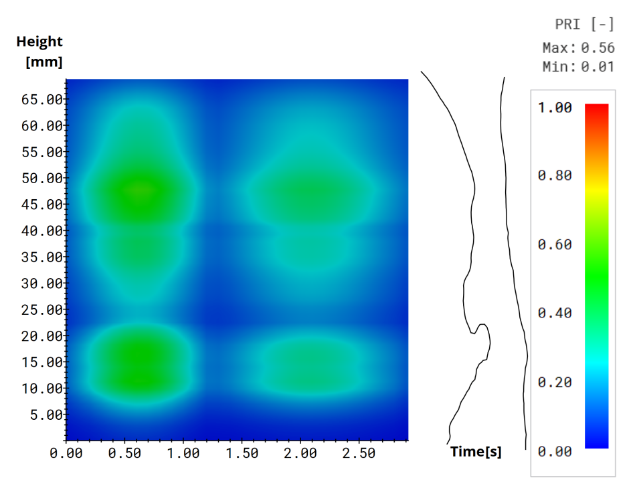 | 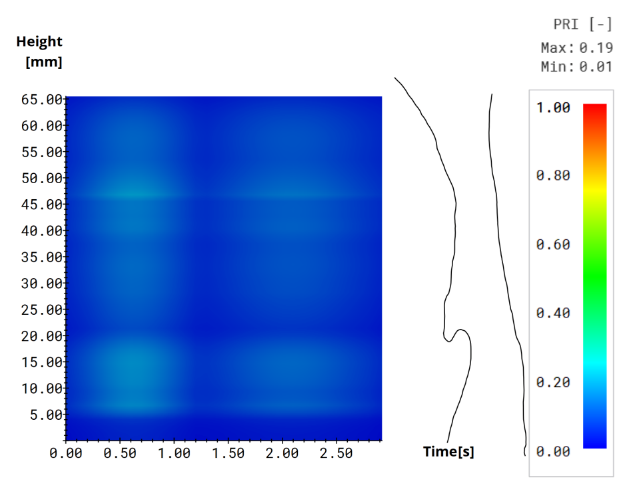 |
| 6 | 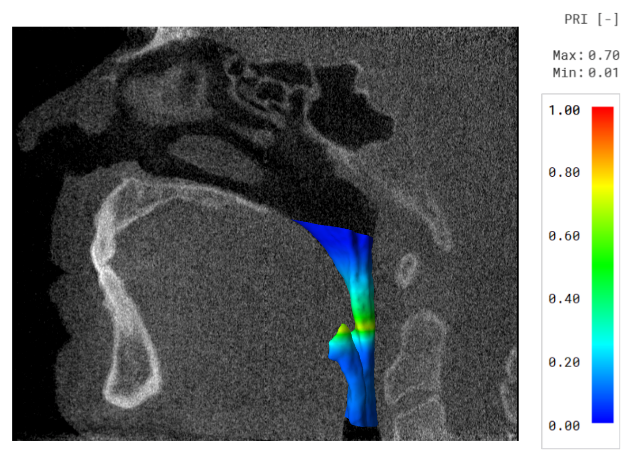 | 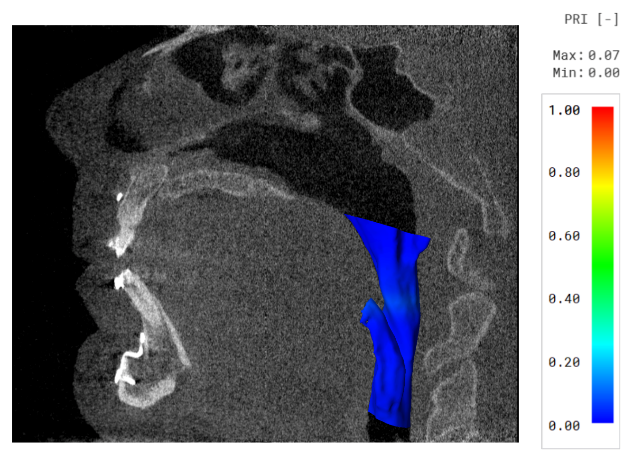 |
|  | 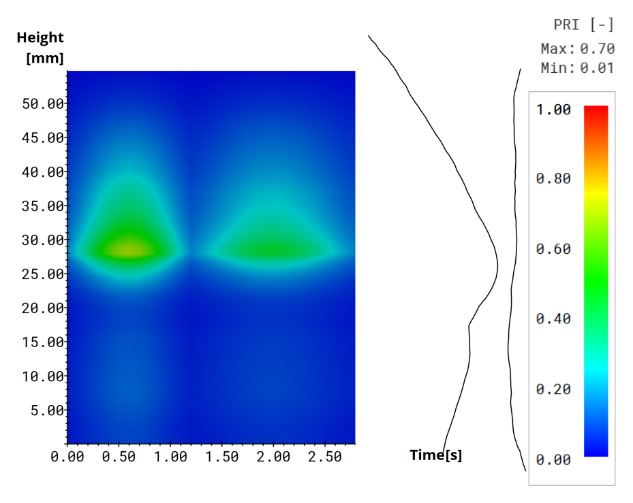 | 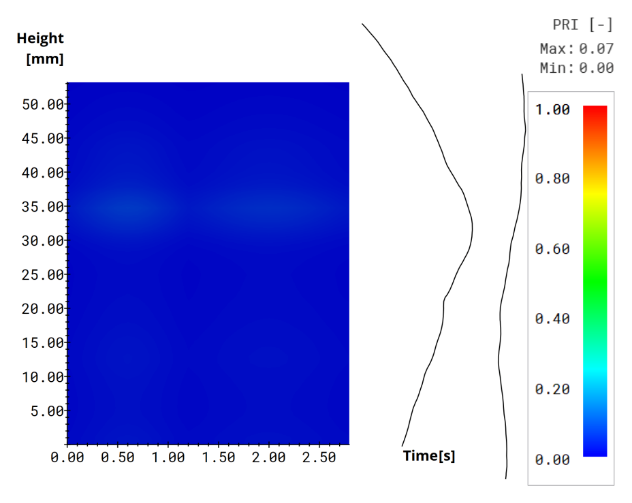 |
| 7 | 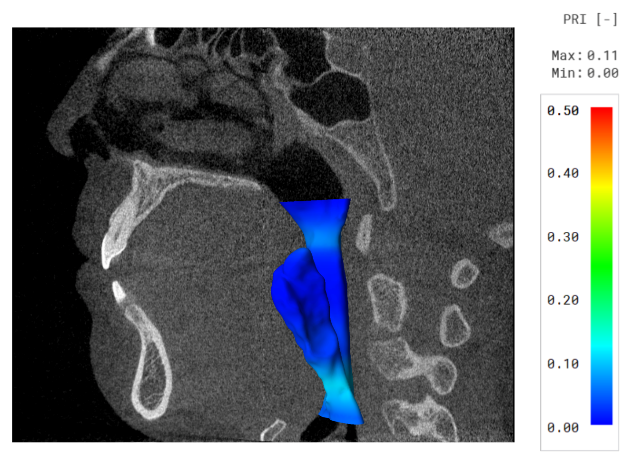 | 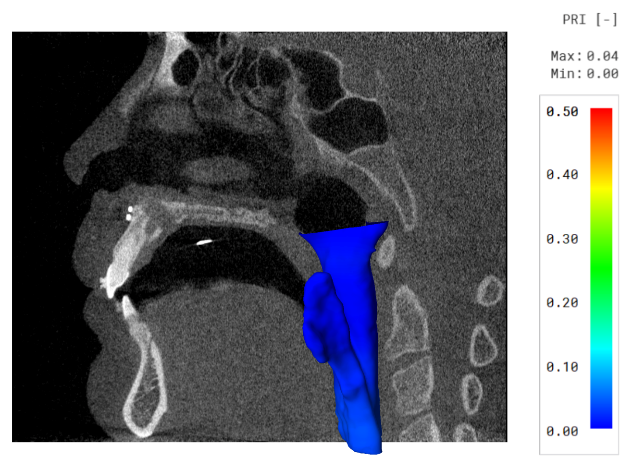 |
|  | 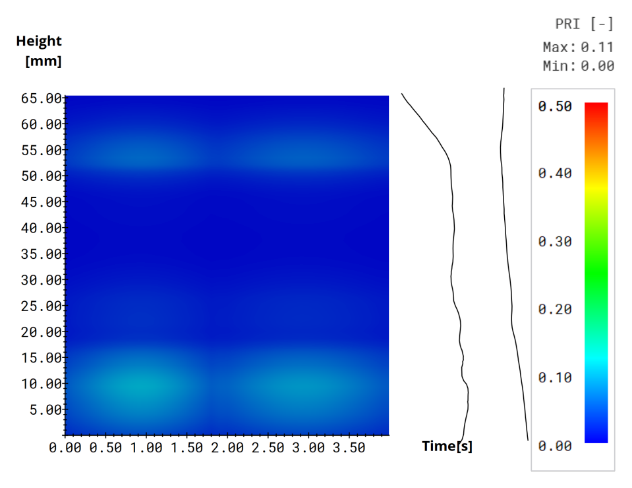 | 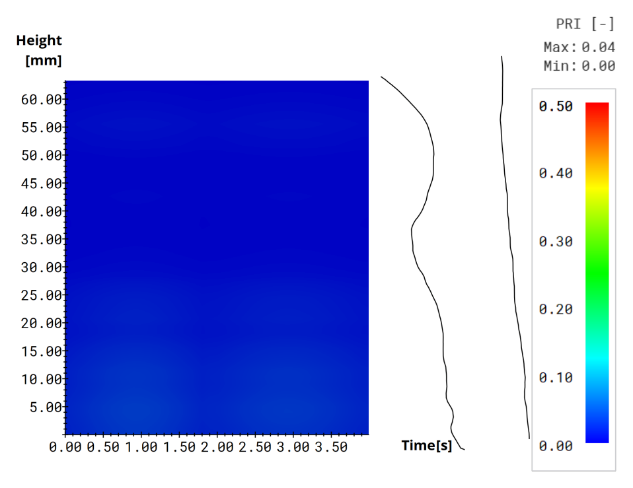 |
| 8 | 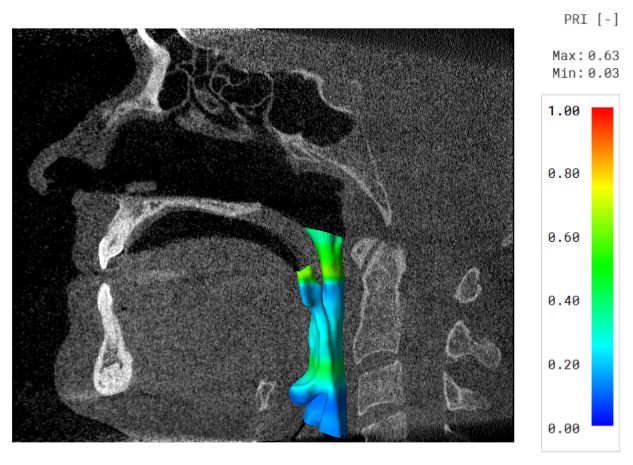 | 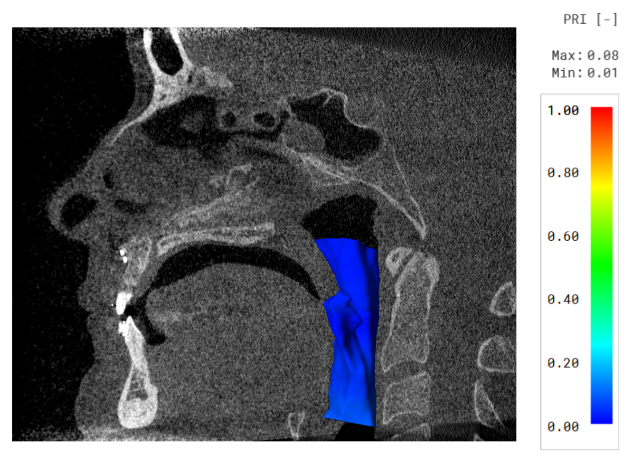 |
|  | 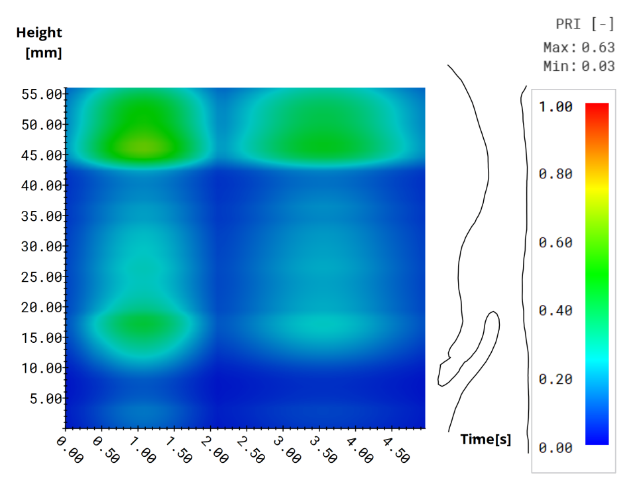 | 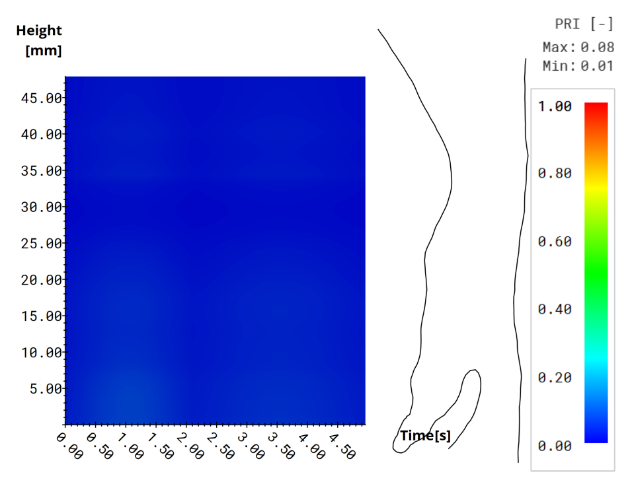 |
| 9 | 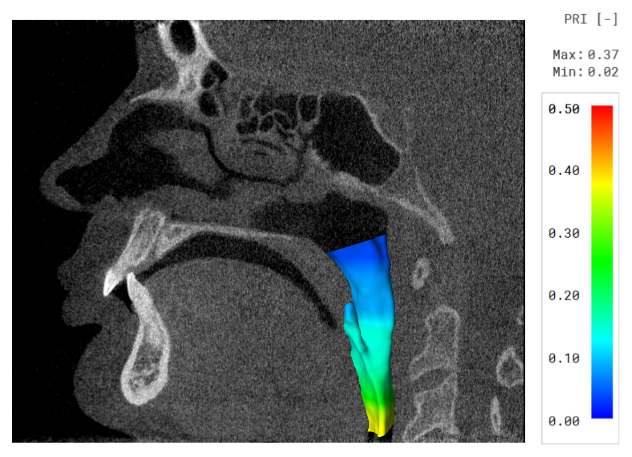 | 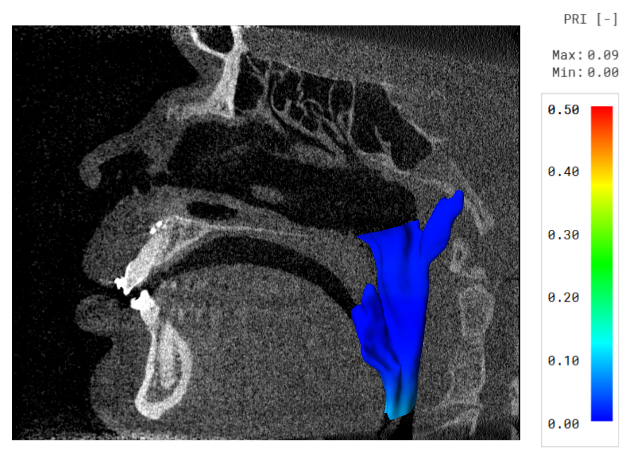 |
|  | 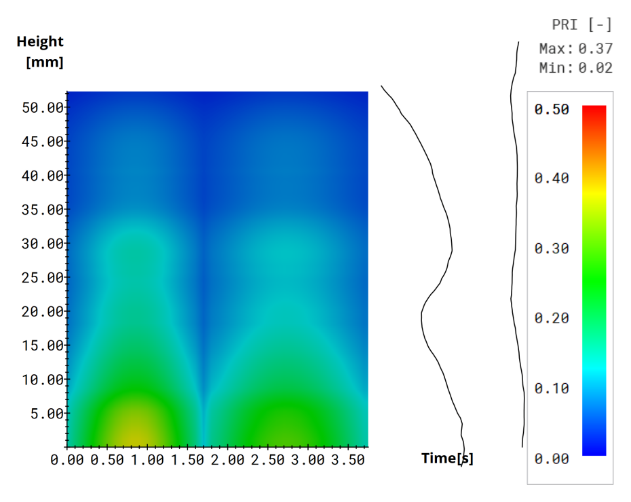 | 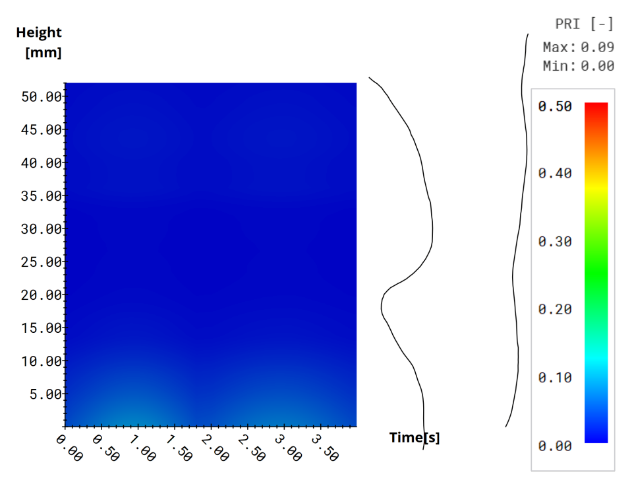 |
| 10 | 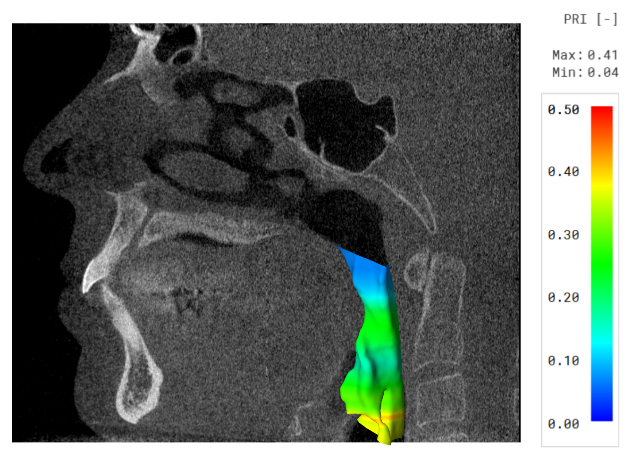 | 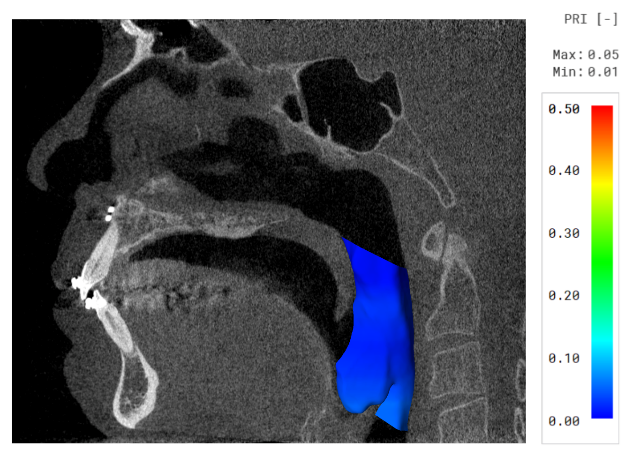 |
|  | 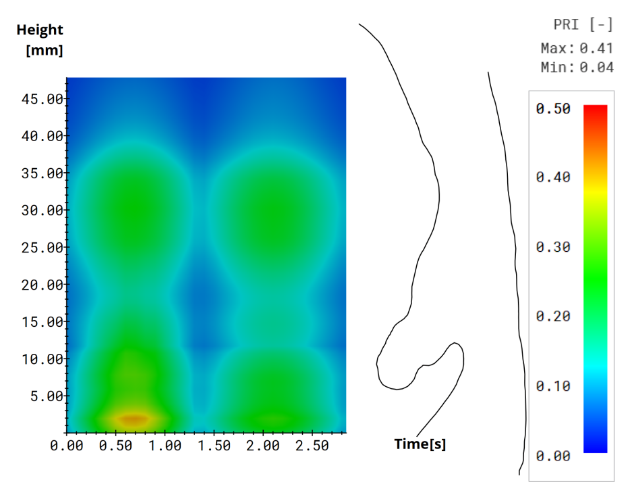 | 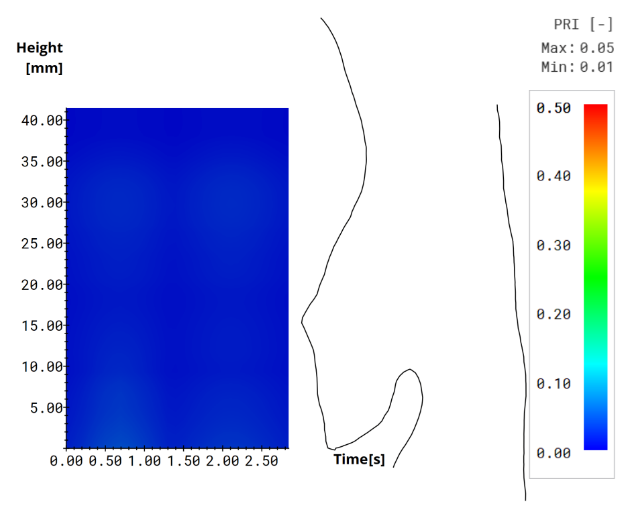 |
| 11 | 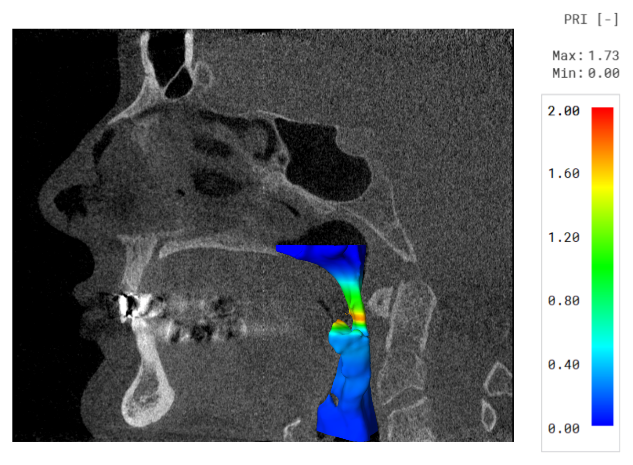 | 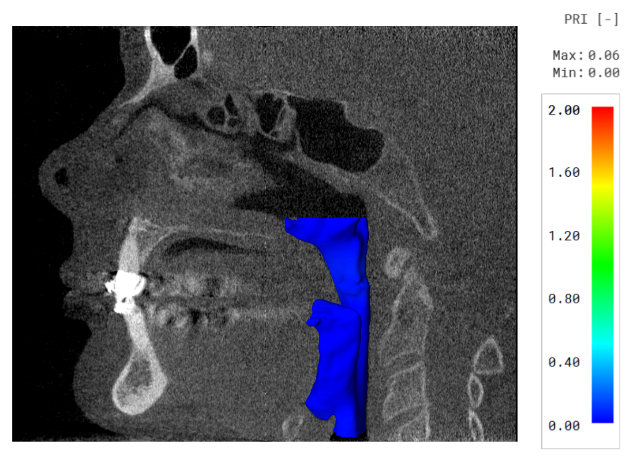 |
|  | 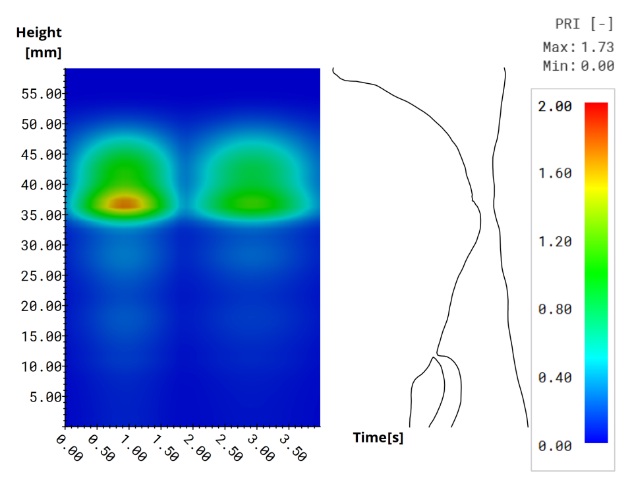 | 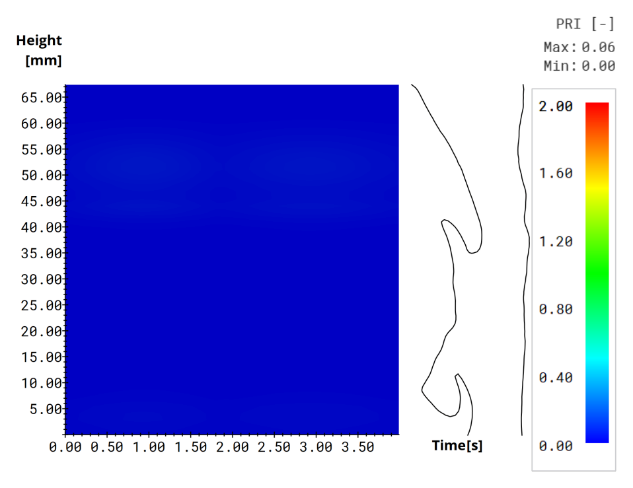 |
| 12 | 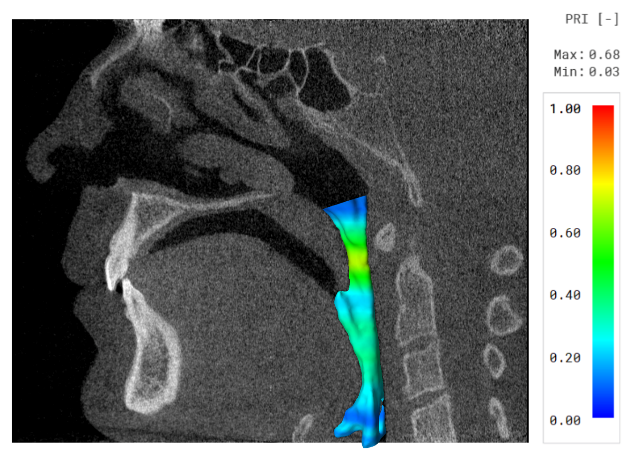 | 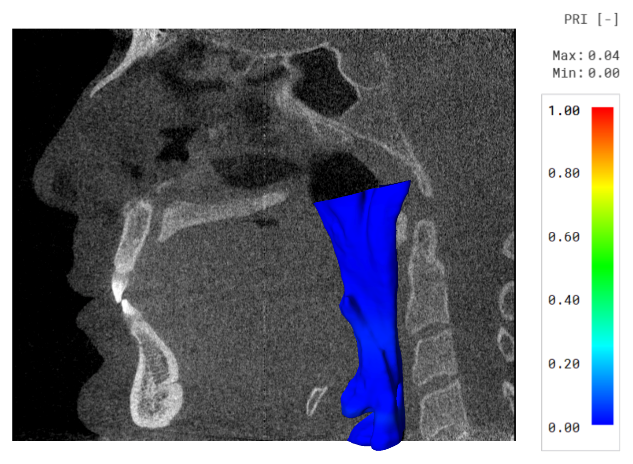 |
|  | 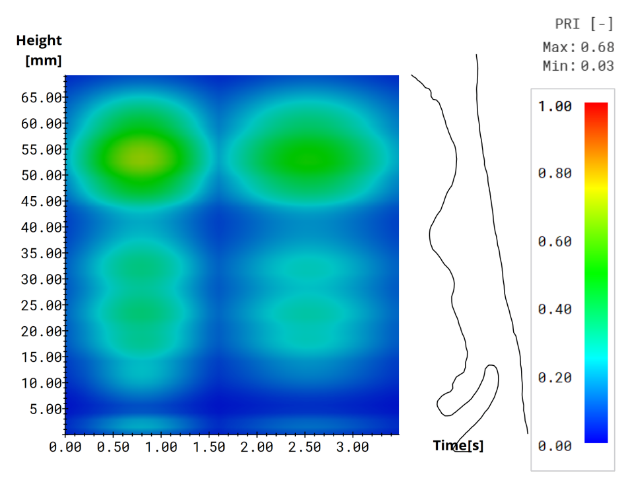 | 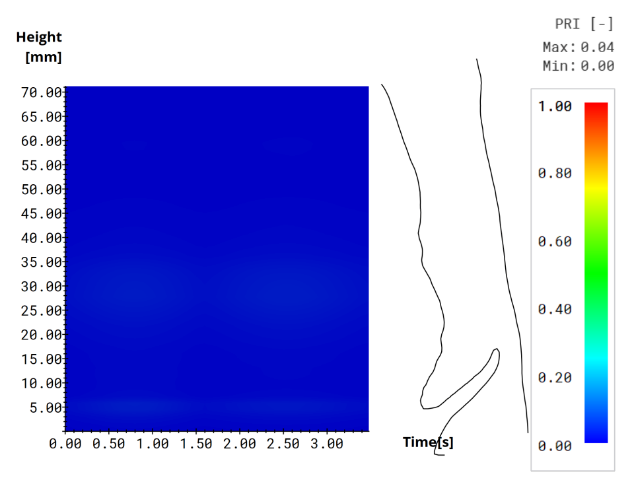 |
| 13 | 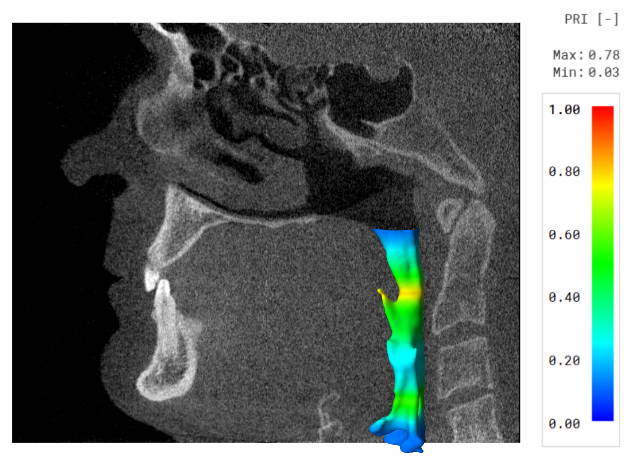 | 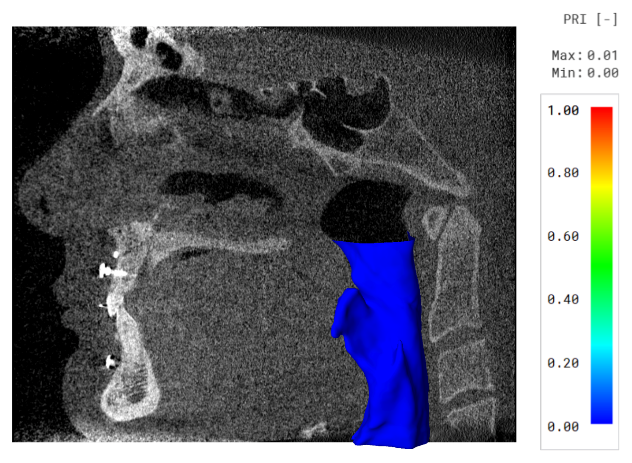 |
|  | 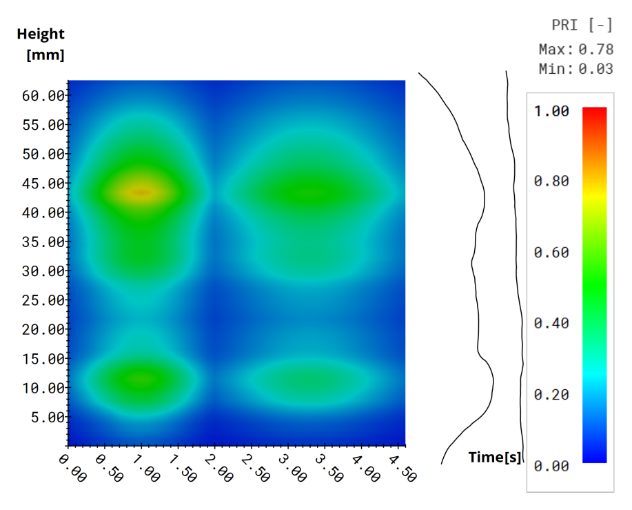 | 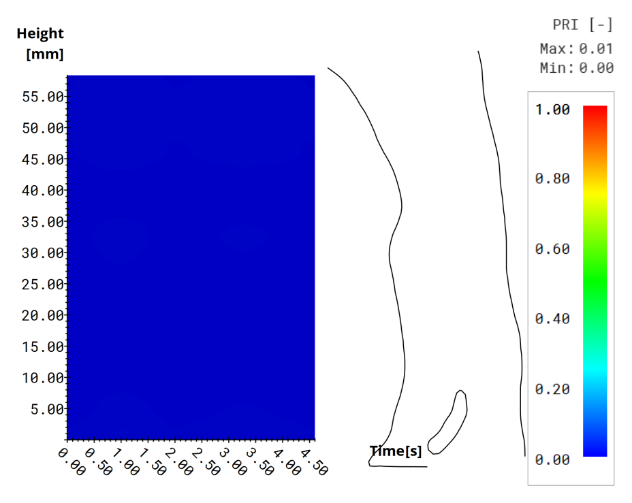 |
| 14 | 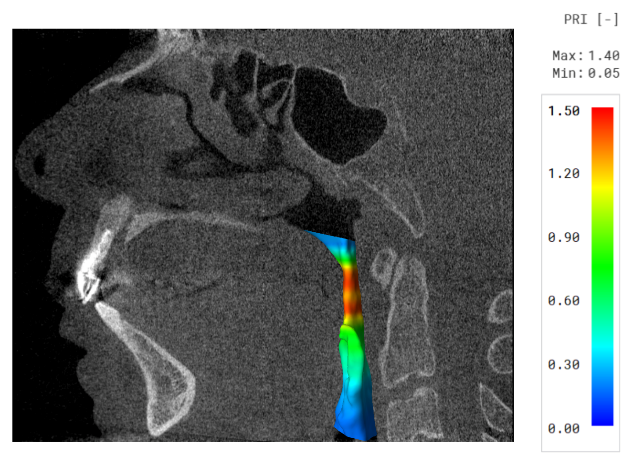 | 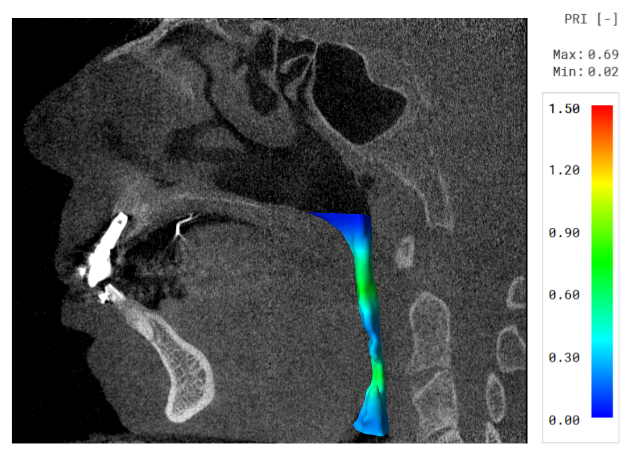 |
|  | 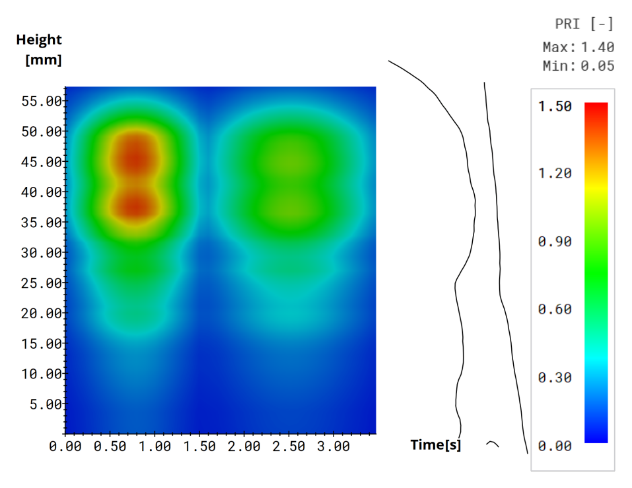 | 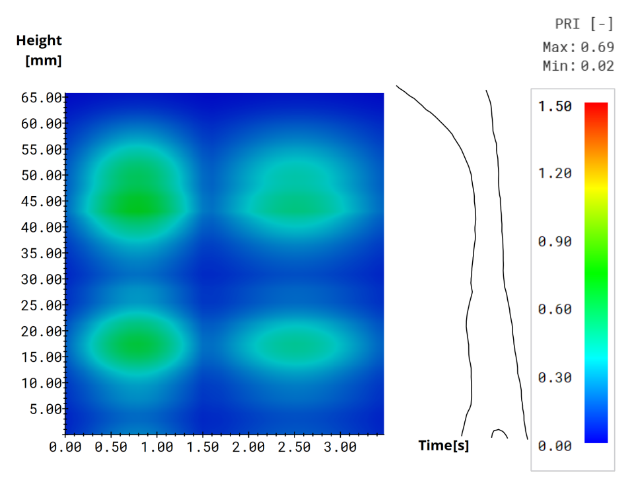 |
| 15 | 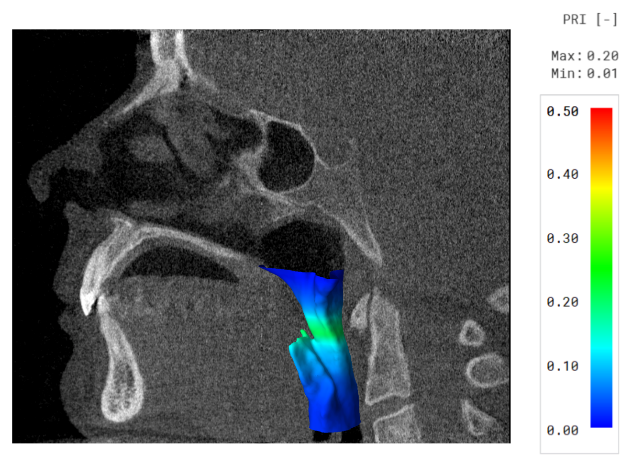 | 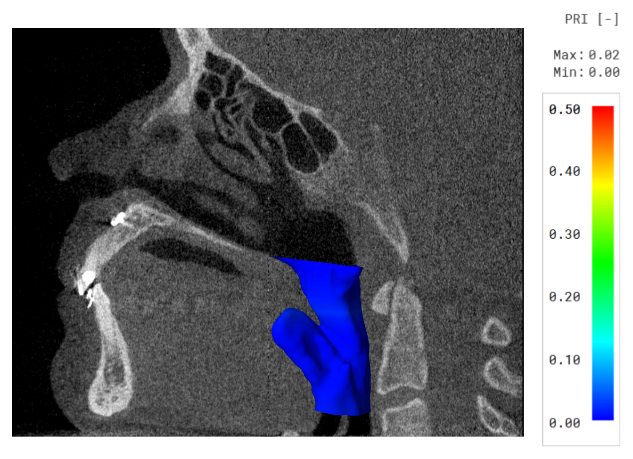 |
|  | 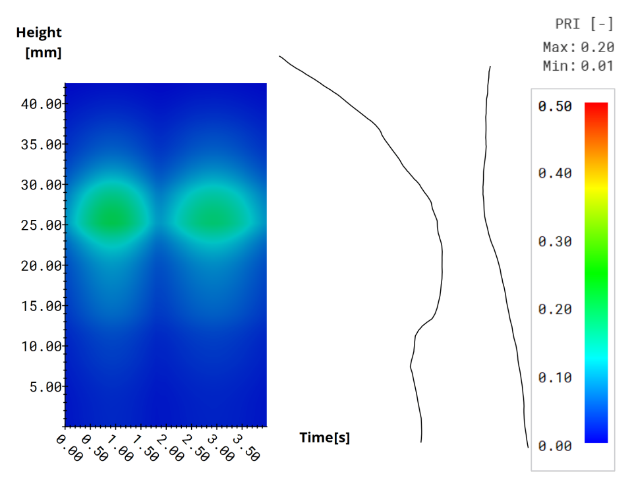 | 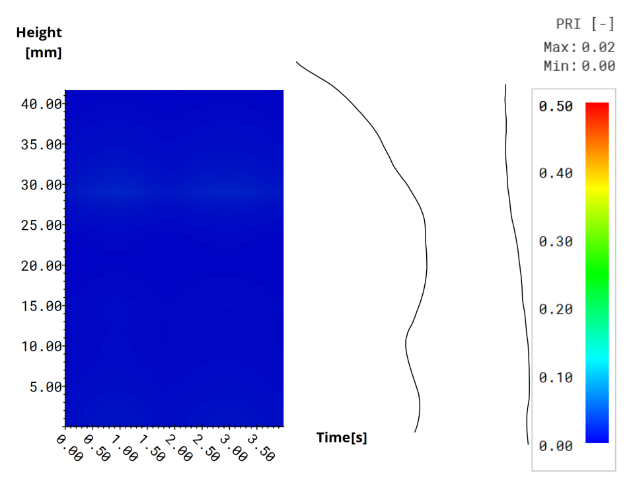 |
| 16 | 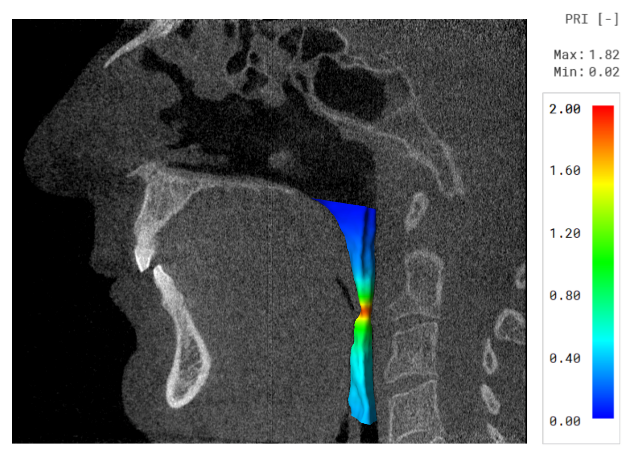 | 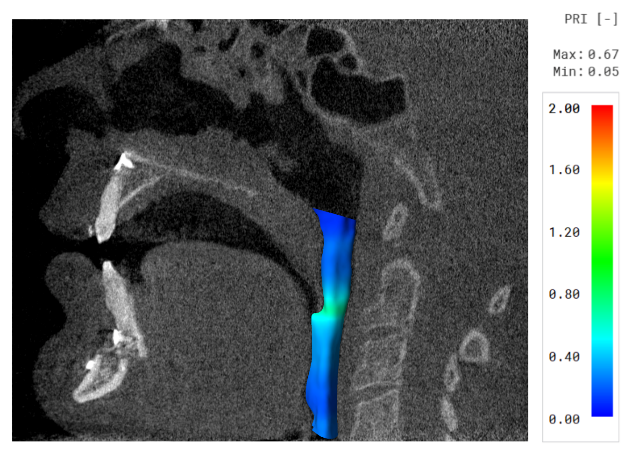 |
|  | 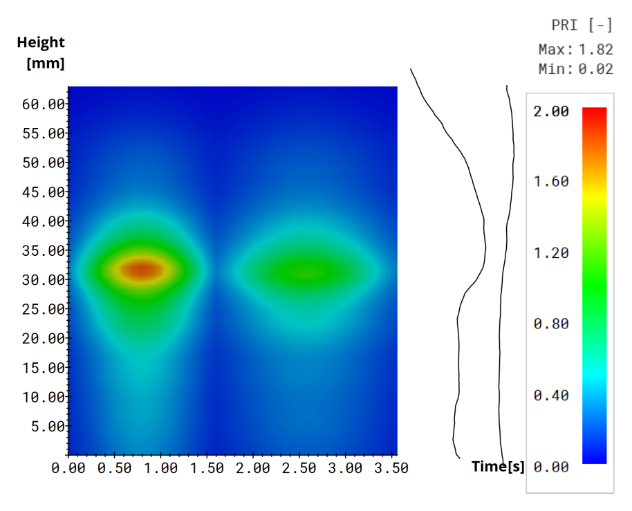 | 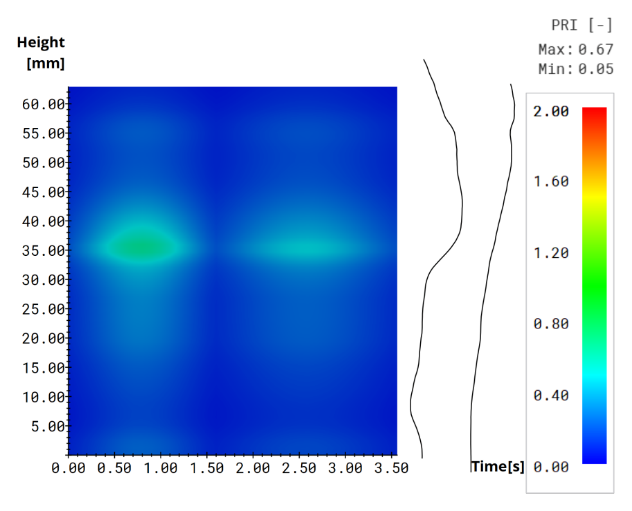 |
